# Spectroscopic (FT-IR and FT-Raman) and quantum chemical study on monomer and dimer of benznidazole from DFT and molecular docking approaches

**DOI:** 10.1016/j.heliyon.2025.e42104

**Published:** 2025-01-20

**Authors:** Tirth Raj Paneru, Manoj Kumar Chaudhary, Poonam Tandon, Bhawani Datt Joshi, Beatriz Pinheiro Bezerra, Alejandro Pedro Ayala

**Affiliations:** a Central Department of General Science, Far Western University, Mahendranagar, 10400, Nepal; b Central Department of Physics, Tribhuvan University, Kirtipur, Kathmandu, Nepal; c Department of Physics, Tribhuvan University, Amrit Campus, Institute of Science and Technology, Kathmandu, 44600, Nepal; d Deen Dayal Upadhyaya Gorakhpur University and University of Lucknow, Lucknow, 226007, India; e Department of Physics, Tribhuvan University, Siddhanath Science Campus, Mahendranagar, 10400, Nepal; f Department of Physics, Federal University of Ceará, Fortaleza, CE, 60440-900, Brazil

**Keywords:** Molecular docking, Benznidazole, Natural bond orbital, Reduced density gradient, PDOS, Vibrational spectra

## Abstract

This work presents the quantum chemical calculations of the monomer and dimer of benznidazole using density functional theory (DFT) at the B3LYP/6−311++G(d,2p) level of theory. A one-dimensional potential energy surface scan was carried out across flexible bonds to find the minimum energy structure. The structure with minimum energy was taken as a monomer and dimer is constructed based on intermolecular hydrogen bonding N−H**…**O. The vibrational analysis was conducted by comparing the calculated FT-IR and FT-Raman spectra of the monomer and dimer with the experimental ones. The red shift in the spectra of amide and carbonyl functional groups indicates their involvement in intermolecular hydrogen bonding in crystal packing, while the other peaks showed good agreement with the experimental result. The intra- and intermolecular interactions in the monomer and dimer were analyzed using various tools. The steric effects and van der Waals forces in the dimer were found to be more effective than the monomer. The dimer in the gaseous medium was found to have a lower Frontier molecular orbital energy (ΔE_L-H_) value than the monomer, suggesting that it is more reactive in a gaseous medium. The ELF value for hydrogen in monomer and dimer around the ring was found to be more which confirms that the electrons in these regions are more localized. The negative value of the overlap population density of states (OPDOS) both in monomer and dimer indicate that there are anti-bonding orbitals between the acetamide and the benzyl groups of the compound. The drug potential of benznidazole was evaluated by molecular docking with carbonic anhydrase XII, which shows the highest binding affinity of (−8.3 kcal/mol) with 6YH8, indicating that benznidazole is its potent inhibitor.

## Highlights

• Geometry optimization of monomer and dimer of benznidazole have been performed.
• AIM study and NCI analysis for monomer and dimer have been carried out.
• Molecular stability and reactivity were scrutinized using NBO analysis.
• Experimental and calculated vibrational spectra were simulated.
• Drug likeness and docking of benznidazole with carbonic anhydrase XII has been done.

## Graphical abstract

**Figure:**
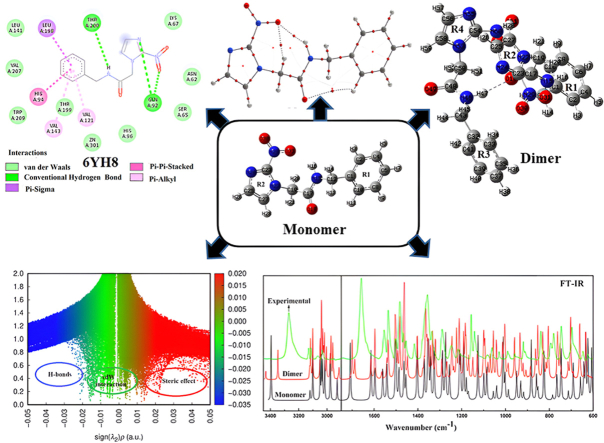

## 1 Introduction

Nitroimidazoles are active compounds which is used to treat protozoan infections. Benznidazole (N-benzyl-2-(2-nitroimidazol-1-yl)acetamide) is an active compound of the nitroimidazole class, formed by adding a nitro group to the imidazole rings [1]. Benznidazole (C_12_H_12_N_4_O_3_) is a monocarboxylic acid that is an antiprotozoal drug. It contains an imidazole group, an acetamide fragment, and a benzyl group. The strong intermolecular hydrogen bonds between the amide group (NH) and the carbonyl group (C=O) help in crystal packing [2]. Benznidazole is used to treat Chagas disease, which is caused by the protozoan parasite *Trypanosoma cruzi* [3]. The parasite that causes Chagas disease is transmitted by triatomine-contaminated food, drug users, donations of organs, transmission from mother to child, blood donations, and by the kissing of triatomine bugs [4,5]. Benznidazole and nifurtimox are two drugs available for the treatment of Chagas disease for urgent use [6]. Triazole derivatives are also being studied in clinical trials to treat Chagas disease in its chronic phase [7]. Despite ongoing clinical trials, no new drug type has shown a safe profile that outperforms nitroimidazoles regarding treatment results [8]. Benznidazole reduces parasite loads in tissues, which reduces parasitemia and maternal Chagas disease transmission [[9], [10], [11], [12]]. The hydrogen bonding in the molecule is responsible for the conformational changes and performance of biological activity [13]. The solubility of compound has been tested and its value is found to be increased in three polymorphs of a cocrystal of benznidazole and salicylic acid [14]. Boron and carbon nanotubes were used as potential benznidazole carriers to improve solubility, and it was found that boron nanotubes were superior to carbon nanotubes [15]. Cocrystal screening of benznidazole via hydrogen bonding with the former containing carboxylic group investigated that O−H…N hydrogen bonding is more beneficial than C−H…O interaction [16].

**Fig. 1:**
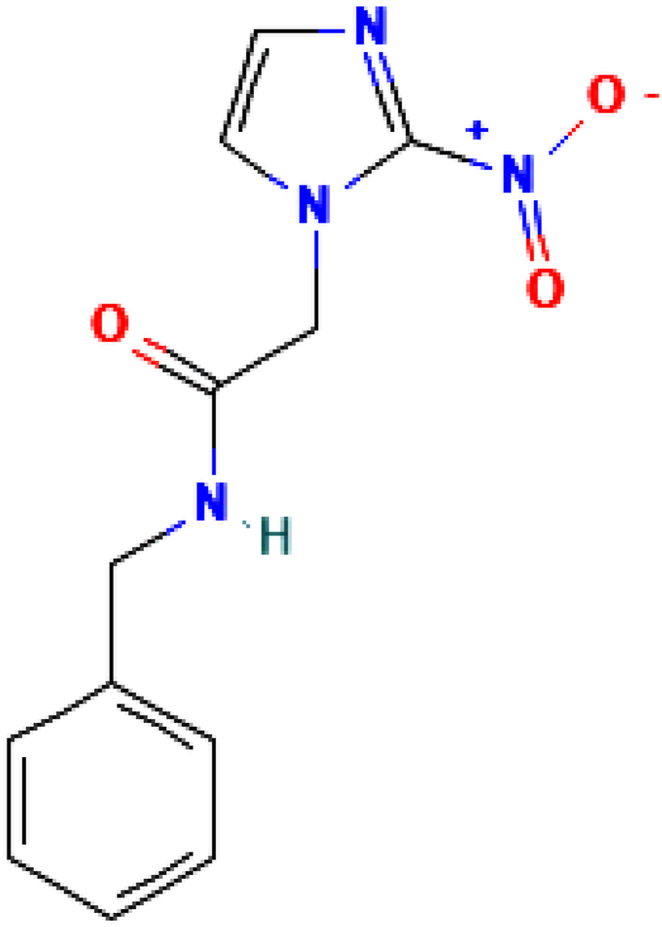
Chemical structure of benznidazole.

The literature reveals that the comparative study of monomers and dimers, considering their electronic properties as well as the path of biological activity through quantum chemical calculation, has not been recorded yet. This study aims to treat the monomer and dimer of the title molecule with DFT and provide a thorough understanding of intra- and intermolecular hydrogen bonding in a variety of aspects by clarifying reactive sites, reactivity, stability, and charge transfer properties. The vibrational spectra, Fourier Transform infrared (FT-IR) and Fourier Transform Raman (FT-Raman) of monomers and dimers were investigated using quantum chemical calculations and compared to experimental data. QTAIM was used to examine the topology of electron density distributions around atoms and along bond paths, revealing information about intra- and intermolecular hydrogen bonds. Electron localization function (ELF) was used to analyze electron localization in an atom or molecule. The reduced density gradient (RDG) plot illuminates non-covalent interaction (NCI) and provides a comprehensive understanding of intra- and intermolecular interactions in a molecular system. The natural bond orbital (NBO) analysis was also used to examine the stability of molecules and intermolecular hydrogen bonding. Reactive sites for biological activities have been identified and chemical reactivity and stability has been investigated using molecular electrostatic potential (MEP) mapping, the values of ΔE_L-H_, global and local reactivity descriptors. Moreover, the composition of molecular orbitals and their impact on chemical bonding were revealed by total density of state (TDOS), partial density of state (PDOS), and OPDOS, demonstrating how different orbitals contribute to the overall electronic structure and bonding characteristics within molecules [17]. The thermodynamic properties of a molecule determine how it responds to temperature changes, whereas drug-like properties evaluate a molecule's potential as a pharmaceutical agent. Molecular docking was utilized to predict the binding strength of benznidazole with the specific protein code of carbonic anhydrase XII to assess its biological performance. This also identifies the reactive sites of the title molecule for its biological activity. The chemical structure of benznidazole is depicted in Fig. 1.

## 2 Materials and methods

### 2.1 Experimental work

Benznidazole was kindly donated by Laboratório Farmacêutico do Estado de Pernambuco (LAFEPE) and used without further purification. The FT-IR spectrum was recorded at room temperature using a Bruker Vertex 70 spectrometer equipped with an Attenuated Total Reflectance (ATR) accessory based on a ZnSe crystal. The absorbance spectra were recorded in the region of (3600 ‒ 600) cm^−1^ with a resolution of 2 cm^−1^. The FT-Raman spectra were acquired on a LabRAM HR (Jobin-Yvon HORIBA) spectrometer, equipped with a charge-coupled device (CCD device, charge-coupled) cooled with liquid nitrogen. A HeNe laser operating at 632.8 nm wavelength was used for the excitation of the samples. The laser beam was focused on the sample surface using a microscope (OLYMPUS) with a lens of 50x and numerical aperture of 0.75 forming a spot of approximately 4 μm over the surface of the sample.

### 2.2 Computational details

The optimization procedure of compound has been carried out by using the Gaussian 16 software package [18,19]. The 6−311++G(d,2p) basis set employed the B3LYP hybrid functional, which combined Becke's three exchange parameters with the correlation functional of Lee, Yang, and Parr. This hybrid functional B3LYP enhances accuracy for bond lengths, vibrational frequencies, and energies by utilizing a basis set with additional diffuse and polarized functions for a more precise description of electron behavior [[20], [21], [22], [23]]. The optimized structure, the molecular charge distribution in the MEP map, and the orbital lobs are visualized by GaussView 06 [24]. A one-dimensional potential energy surface (PES) scan was conducted to determine the most stable conformers. The QTAIM method was applied to study the topology of electron density using the AIMALL (10.05.04) software package [25,26]. The RDG scatter plots and isosurface were analyzed for NCI and plotted using VMD 1.9.4 and Multiwfn 3.8 software [27,28]. Multiwfn 3.8 software was also employed to examine the TDOS, PDOS, and OPDOS. Additionally, the NBO analysis of donor-acceptor interactions within molecules was carried out through the NBO 3.1 program, which is a part of the Gaussian 16 software [29]. AutoDock Tools (1.5.4) was used to perform molecular docking between benznidazole and carbonic anhydrase XII, and Discovery Studio Visualizer 4.5 was used to visualize the binding sites [30,31].

## 3 Results and discussion

### 3.1 Conformational analysis

**Fig. 2:**
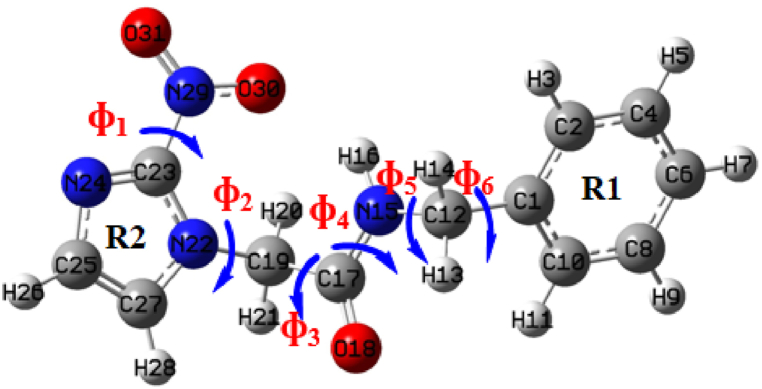
Optimized structure of benznidazole (arrow represent the flexible bonds).

**Fig. 3:**
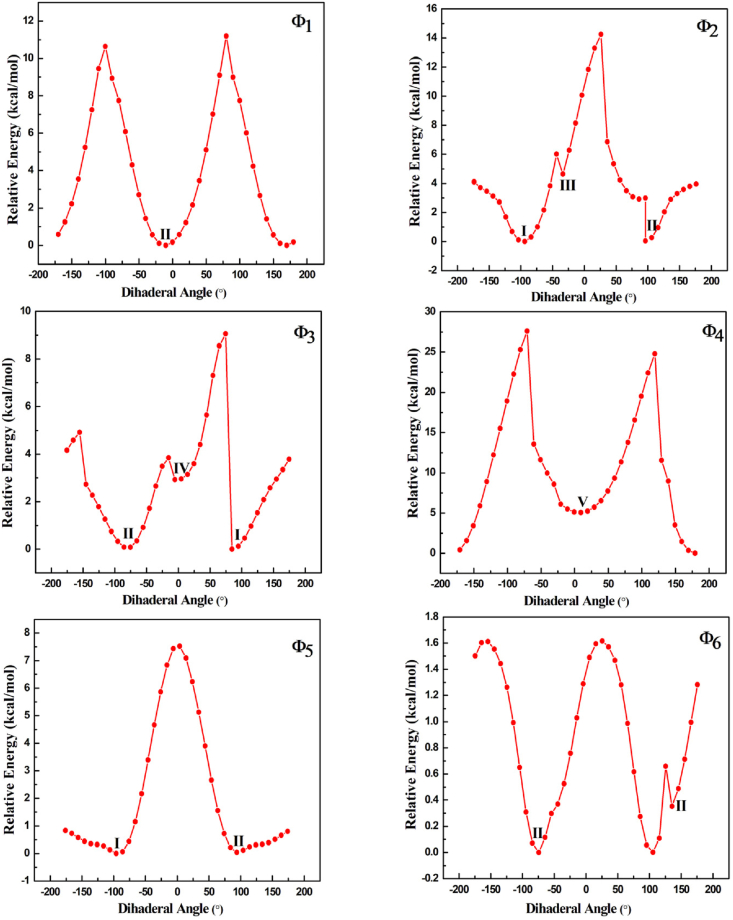
Scan graphs showing the variation of the relative energy of benznidazole with the dihedral angle at flexible bonds.

Computational analysis identified two distinct conformers, s-trans and s-cis, and noticed that internal hydrogen bonds are responsible for structural variations in benznidazole [32,33]. In this study, the molecule was optimized at the B3LYP/6−311++G(d,p) level of theory, and one-dimensional PES scan was conducted across the flexible bonds C23-N29, N22-C19, C19-C17, C17-N15, N15-C12, and C12-C1, to find most stable conformer as depicted in Fig. 2. The variation of relative energy with the dihedral angle, with a variation of 10° in each step, is shown in the scan graph given in Fig. 3.

**Fig. 4:**
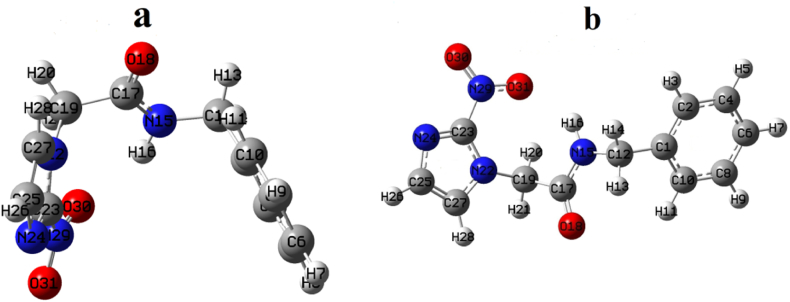
Stable conformers: (a) conformer I, and (b) conformer II of benznidazole (obtained from B3LYP/6−311++G(d,2p) level of theory).

The molecule was further optimized using B3LYP/6−311++G(d,p) and B3LYP/6−311++G(d,2p) levels of theory, at local and global minima to find the most stable conformer. Altogether, five conformers were obtained, and their respective energies are listed in Table S1 (supplementary material). Conformers with a relative energy difference of less than 0.56 kcal/mol (equivalent to kT) are more probable [34], so two stable conformers of benznidazole have been identified as conformer I and conformer II, as shown in Fig. 4(a and b), respectively. The energy of the stable conformer with the 6−311++G(d,2p) basis set was reduced by 3.19 kcal/mol compared to the 6−311++G(d,p) basis set, which is demonstrated in Table S2. This reduction in energy is caused by a higher level of the polarization function '2p′, which provides more accurate and stable molecular electronic structure [35]. The structures of all the conformers and their relative energy concerning minimum energy structure (conformer I) are shown in Fig. S1. The most stable conformers were also optimized using the WB97XD functional. When the B3LYP functional was used, the ground state energy of the most stable conformer was found to be lower than that of Functional WB97XD, as shown in Table S2. So, further calculations were performed with the B3LYP/6−311++G(d,2p) level of theory. The benchmark report claims B3LYP performs better in electronic and spectroscopic results than WB97XD [36].

**Fig. 5:**
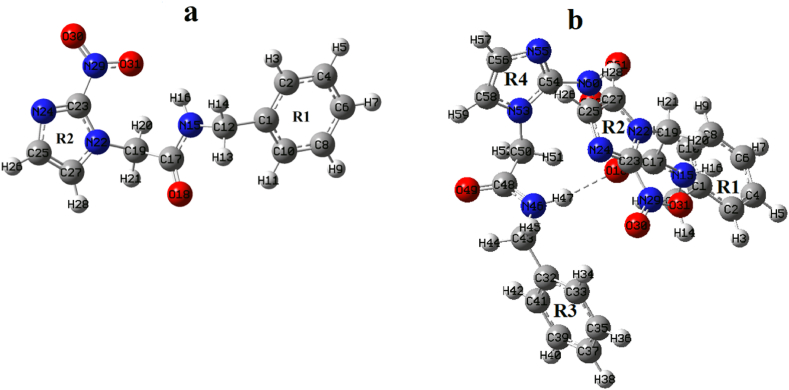
Optimized structures: (a) monomer and (b) dimer of the benznidazole.

The overlapping of crystallographic structure (CCDC 680687) with the stable conformers of benznidazole has been done using Molsoft software and was displayed in Fig. S2 [37,38]. Conformer II exhibited remarkable agreement with the crystal structure, with the lowest value of RMSD (0.70 Å) in comparison to other structures, so this conformer was selected as the stable monomer. The intermolecular interaction between NH (amide) and C=O (carbonyl) groups results in the formation of dimers. The optimized structures of the monomer and dimer of the title compound are shown in Fig. 5(a and b), respectively. The interaction energy forming dimer was found to be −15.72 kcal/mol, obtained by the difference between the calculated energy of the dimer and the sum of the calculated energy of monomers, by the contribution of hydrogen bonding N46-H47 … O18. The structure of the dimer is greatly influenced by the basis set superposition error (BSSE). The energy of the dimer with counterpoise correction was determined to be −13.21 kcal/mol with a BSSE correction of 2.51 kcal/mol.

### 3.2 Optimized structure parameters and AIM study

The optimized structural parameters, including bond lengths and angles compared with XRD crystal structure data by Soares Sobrinho et al. [2] for the monomer and dimer of benznidazole, are listed in Table S3. The least value of RMSD (0.077 Å) of bond lengths for monomer and dimer with XRD data signifies a good correlation between them. The RMSD value of bond angle is 1.610° between calculated and XRD structure data which indicates a high degree of agreement between them. The calculated bond lengths for C8-H9, N15-H16, N15-C17, N22-C23, N29-O30, and N29-O31 differ from crystal structure data by 0.130, 0.170, 0.023, 0.015, 0.012, and 0.011 Å in monomer and 0.130, 0.170, 0.015, 0.013, 0.007, and 0.006 Å in dimer, respectively. The difference between the XRD crystal structure data and calculated values across bonds C34-H40, N46-N47, N46-C48, N53-C54, N60-O61, and N60-O62 was found to be 0.130, 0.180, 0.020, 0.009, 0.001, and 0.00 Å. The calculated bond angles differ from XRD crystal structure data across C1-C12-H13, C1-C12-N15, H13-C12-N15, C12-N15-H16, H26-C25-C27, and C25-C27-H28 by 1.1, 2.2, 2.9, 0.8, 3.4, and 5.7° in the monomer and 1.8, 0.9, 3.1, 2.4, 3.3, and 5.5° in the dimer. The difference between the XRD data and the calculated data was found to be 1.6, 1.3, 3.0, 1.8, 3.3, and 6.1° for angles C32-C43-H44, C32-C43-N46, H44-C43-N46, C43-N46-H47, H57-C56-C58, and C56-C58-H59 in the dimer. The presence of intermolecular hydrogen bonding in the crystal packing causes some of the mentioned bond lengths and bond angles for the calculated structure to differ from crystal structure data. The dihedral angle O18-C17-N15-H16 involves both NH and C=O groups, which participate in intermolecular hydrogen bonding in the solid-state structure. This angle measures approximately −170.7° in the XRD structure, while in the optimized structure of the monomer, it is 179.1°. These variations may play a crucial role in conformational changes and the overall geometry of the structure.

**Fig. 6:**
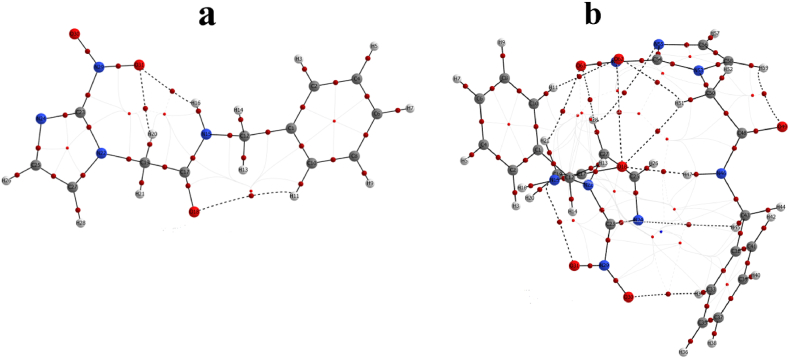
Molecular graph showing intra- and intermolecular hydrogen bonding in (a) monomer, and (b) dimer of the benznidazole.

The intra- and intermolecular hydrogen bonding in monomer and dimer benznidazole molecules was also examined through QTAIM analysis [39]. The topological parameters and geometrical parameters for intra- and intermolecular interaction in the monomer and dimer of the title molecule are presented in Table 1. The comparative hydrogen bond geometry of DFT and XRD structure for intra-molecular interaction in monomer and intermolecular interaction in the dimer is presented in Table S4. For hydrogen bonding, the electron density should be in the range (0.002−0.034) a.u, and the Laplacian lies within (0.024−0.139) a.u [40]. There are many intra-molecular and intermolecular hydrogen bonds in the monomer and dimer of benznidazole, which is shown in the molecular graph in Fig. 6(a and b), respectively. For all intra-molecular and intermolecular hydrogen bonding in monomer and dimer satisfying the conditions Laplacian of electron density ($\nabla^{2}$ ρ_BCP_) > 0 and total electron density (H_BCP_) < 0, this shows a medium hydrogen bond with a partial covalent character in monomer and dimer [41]. The calculated interaction energy for H20 … O31 in the monomer and H47 … O18 in the dimer is large, with the least bond length as compared to other hydrogen bonding, indicating that these bonds make strong interactions. The strong intermolecular hydrogen bond N46-H47 … O18 created the benznidazole dimer. In the crystal structure, the hydrogen bond H47 … O18 was found to have a length of 2.037 Å, while in the optimized dimer structure, it was 1.964 Å, showing good agreement. The intermolecular hydrogen bonding angle N−H…O in the XRD crystal structure is 160.2°, while it was found to be 167.9° for the optimized structure of the dimer [2]. Higher bond ellipticity for H11 … O18 in monomer and for H45 … N24 and H59 … O49 in dimer was found to be higher in comparison to other hydrogen bonding; hence, the electron density distribution at these bond critical points (BCP) is anisotropic, and these bonds are weaker as compared to others [42]. The total of all the energies involved in intermolecular interactions is the interaction energy determined by the AIM calculation [43]. It was discovered that the calculated interaction energy was −11.94 kcal/mol, which is almost equivalent to the interaction energy involved in the formation of the dimer.

**Table 1:** The topological parameters for intra- and intermolecular hydrogen bonding in monomer and dimer of benznidazole.

Mononer								
Interactions	**Bond length(Å)**	**ρ**_**BCP**_	**G**_**BCP**_	**V**_**BCP**_	$\nabla^{2}$**ρ**_**BCP**_	**H**_**BCP**_	**E**_**int**_	**ε**
H16 … O31	3.463	0.01789	−0.00197	−0.01278	0.06686	−0.01475	−4.009	0.0423
H20 … O31	2.424	0.01726	−0.00197	−0.01866	0.06324	−0.02063	−5.856	0.0872
H11 … O18	3.837	0.00394	−0.00062	−0.00231	0.01417	−0.00293	−0.725	1.1417
**Dimer**								
H34 … O30	2.738	0.00498	−0.00057	−0.00293	0.01627	−0.00350	−0.919	0.0552
H45 … N24	3.870	0.00067	−0.00020	−0.00025	0.00264	−0.00045	−0.078	0.7088
H47 … O18	1.964	0.02133	−0.00232	−0.01675	0.08560	−0.01847	−5.255	0.0769
H51 … O18	2.672	0.00632	−0.00088	−0.00392	0.02278	−0.00480	−1.229	0.3906
H11 … O62	2.722	0.00525	−0.00061	−0.00300	0.01691	−0.00361	−0.941	0.0313
H21 … O61	2.492	0.00871	−0.00096	−0.00545	0.02950	−0.00641	−1.709	0.0260
H28 … O61	2.658	0.00664	−0.00074	−0.00396	0.02173	−0.00470	−1.242	0.0113
H28 … N55	3.060	0.00367	−0.00061	−0.00181	0.01208	−0.00242	−0.567	0.0829
H51 … O62	2.254	0.01657	−0.00202	−0.01119	0.06099	−0.01321	−3.511	0.2486
H59 … O49	2.514	0.01023	−0.00130	−0.00630	0.03562	−0.00790	−1.977	1.1050

ρ_BCP_: Electron density at bond critical point (a.u), H_BCP:_ Total energy density (a.u), G_BCP_: Kinetic energy density (a.u), E_int_: Interaction energy (kcal/mol), V_BCP_: Potential energy density (a.u), $\nabla^{2}$ ρ_BCP:_ Laplacian of electron density (a.u), *ε*: Bond ellipticity.

### 3.3 Non-covalent interactions (NCI) analysis

**Fig. 7:**
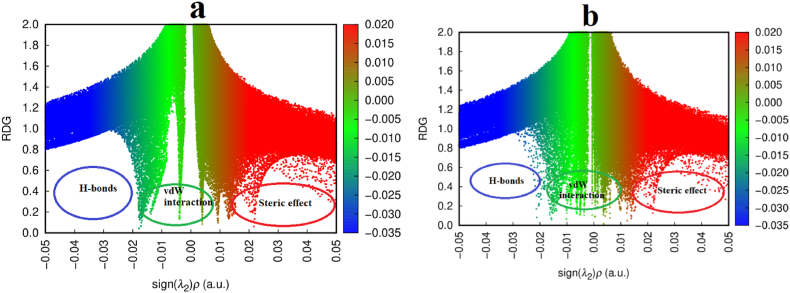
RDG scatter plot for (a) monomer, and (b) dimer of the benznidazole.

**Fig. 8:**
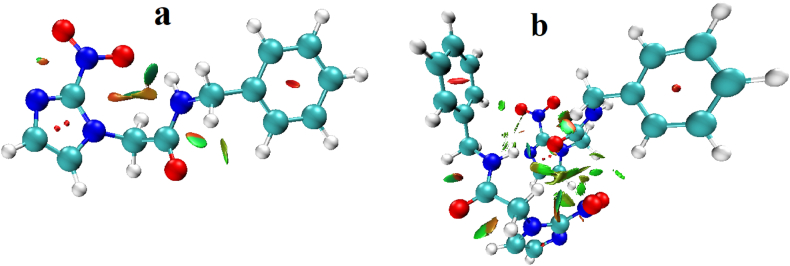
Non-covalent interaction showing in the RDG isosurface (a) monomer, and (b) dimer of benznidazole.

The RDG plot was utilized to visualize numerous NCIs that exist between molecules. The sign of the second largest eigenvalue $\text{sign}\left(\lambda_{2}\right)\rho \left(r\right)$ of a Hessian matrix with electron density is used, which defines a new function, to distinguish between the BCP (3,−1) and ring critical point (RCP) (3,+1) [27,44]. The value $\text{sign}\left(\lambda_{2}\right)\rho \left(r\right)$ can distinguish different types of interactions. RDG is a dimensionless quantity. The strength of the interaction is identified based on the graph plotted between RDG and value of $\text{sign}\left(\lambda_{2}\right)\rho \left(r\right)$ [45]. The negative value of $\text{sign}\left(\lambda_{2}\right)\rho \left(r\right)$ on the left side with blue color represents the region of hydrogen bonding interaction and the positive value of $\text{sign}\left(\lambda_{2}\right)\rho \left(r\right)$ on the right side of the graph with red color is the region of steric effect. The green region with the value $\text{sign}\left(\lambda_{2}\right)\rho \left(r\right)=0$ is the region of van der Waals interactions. The RDG scatter plot with three regions highlighted with red, blue, and green color for the monomer and dimer is shown in Fig. 7(a and b). The color-filled isosurface for the monomer and dimer is presented in Fig. 8(a and b). From the RDG scatter plot and RDG isosurface, we conclude the steric effect was observed around the ring in monomer and dimer. Compared to the monomer, the dimer has a higher likelihood of experiencing the steric effect. Dimer van der Waals interaction is preferred over monomer because there is a greater amount of intra-molecular bonding. Strong intermolecular hydrogen bonding in the dimer is demonstrated by the blue spikes that developed between the H47 and O18; in the monomer, no such spikes were visible.

### 3.4 Electron localization function (ELF) analysis

**Fig. 9:**
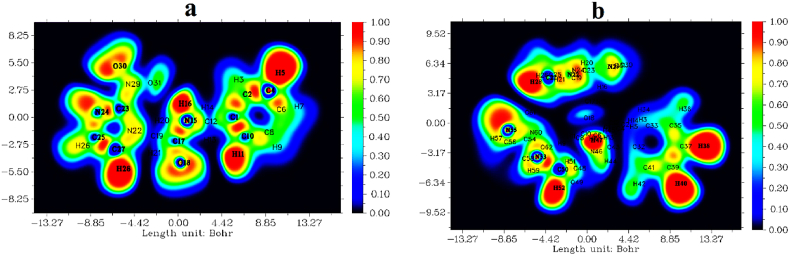
2D-colored filled map for (a) monomer, and (b) dimer of the benznidazole.

**Fig. 10:**
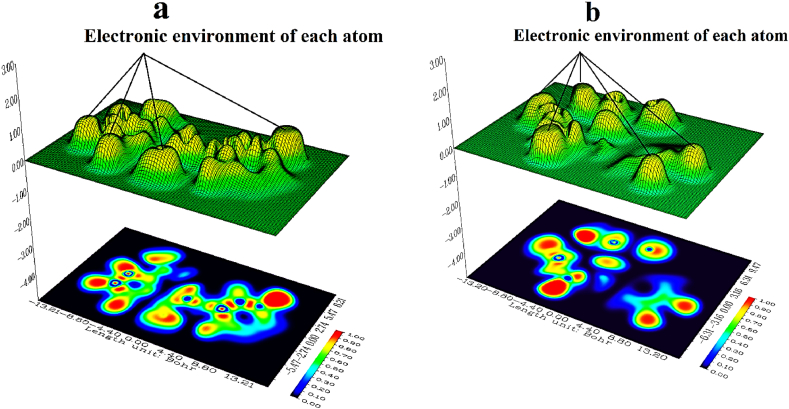
3D projection of the ELF map for (a) monomer, and (b) dimer of benznidazole.

The region of electron localization and electron delocalization was investigated by the ELF. If the ELF is larger, the electrons are isolated from the outside world and their motion is constrained within it [46]. A scale from 0.0 to 1.0 is used to represent the two-dimensional ELF image; red indicates high electron localization, yellow to green indicates moderate localization, and blue shades indicate low electron localization. On the ELF scale, low values indicate less tendency toward electron repulsion, while high values indicate a higher concentration of electrons in a particular area [47]. The two-dimensional color-filled ELF map for the monomer and dimer is displayed in Fig. 9(a and b). The 3D projection-shaded ELF map for both the monomer and dimer of the compound is presented in Fig. 10(a and b). The hydrogen H5, H11, H16, and H28 in the monomer and H28, H38, H47, H48, and H52 in dimer have a red color indicating a higher ELF value; hence electrons are strongly localized in these regions due to the presence of covalent bond. The electronic environment of each atom in dimer and monomer is represented in a 3D projection map. In the image, the carbon atom in monomer and dimer that are surrounded by a blue color, which represents an electron cloud, is not confined to a specific location within a molecular ring. There may be a depletion region between the inner and outer valence layers based on the presence of a blue ring-like pattern surrounding the carbon and nitrogen atoms in the monomer and dimer in the ring.

### 3.5 Vibrational spectra analysis

**Fig. 11:**
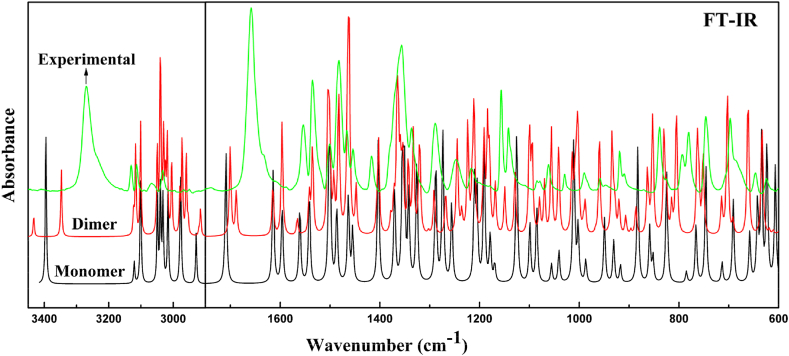
Calculated and experimental IR absorption spectra of monomer and dimer of benznidazole.

**Fig. 12:**
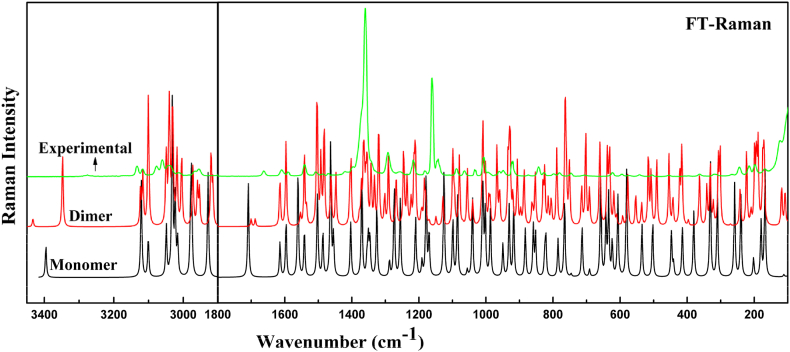
Calculated and experimental Raman scattering spectra of monomer and dimer of benznidazole.

Benznidazole is a monoclinic system, with space group P2_1_ have lattice parameters a = 4.6556(10) Å, b = 10.9113(2) Å, c =11.7681(3) Å, and β =90.6680(10)° with z = 2 [2]. It has 31 atoms and hence gives 87 (3N–6) normal modes of vibration, which are IR and Raman active. In this work, Raman intensities can be computed using the Raman scattering cross-sections, which are proportional to the Raman intensity [48]. To generate simulated spectra, the predicted vibrational mode was convoluted with a Lorentzian line shape with a full width using the computed Raman and IR intensities [49]. Since the calculated wave numbers are higher due to computing anharmonic corrections, hence are scaled down by using a wavenumber scaling procedure (WLS) $[\nu_{obs}=(1.0087-0.0000163\times \nu_{cal}]\nu_{cal}$ [50]. To obtain vibrational modes with their potential energy distribution (PED), using the GAR2PED program, internal coordinates were assigned from the Pulay recommendation [[51], [52], [53]]. Tables S5 and S6 displayed the scaled wavenumbers along with experimental wavenumbers with their PED contribution for the monomer and dimer. The calculated spectrum simulated to match with the experimental FT-IR and FT-Raman spectra is shown in Fig. 11, Fig. 12, respectively.

#### 3.5.1 Vibration of functional groups involved in hydrogen bonding

A strong intermolecular hydrogen bond is formed by the NH group and the C=O group in benznidazole. N−H bond stretching vibrations are commonly observed in hetero-aromatic compounds in the spectral range (3500−3300) cm^−1^ [54]. The stretching vibration of N−H was calculated at 3395 cm^−1^ in the monomer, and it was computed at 3347 cm^−1^ in the dimer. Corresponding observed IR spectra showed peaks at 3277 cm^−1^ and 3270 cm^−1^ in the Raman spectra. In the dimer, the stretching vibration of NH downshifted by 48 cm^−1^ and came closer to the experimental value because it participates in intermolecular hydrogen bonding with the C=O group. The vibrations associated with the C=O group should appear in between 1720 and 1680 cm^−1^ [55]. The stretching vibration for C=O was calculated at 1710 cm^−1^ in the monomer and 1688 cm^−1^ in the dimer. The corresponding observed IR spectra were at 1659 cm^−1^ and 1663 cm^−1^ in the Raman spectra. The calculated spectral wavenumber for the amide and carbonyl group was higher than the observed value, indicating a redshift caused by intermolecular hydrogen bonding in crystal packing. The deviation of spectra in the monomer is more than the dimer from observed data because of the inclusion of hydrogen bonding in the formation of the dimer. The changes in wavenumber in the dimer compared to the monomer due to participation in the hydrogen bond are listed in Table 2.

**Table 2:** Comparison of theoretical and experimental values for the wavenumber and bond lengths of the monomers and dimers in the group of forming hydrogen bonds.

Molecules	NH-group			C=O group		
	Bond length (Å)	IR	Raman	Bond length (Å)	IR	Raman
BNZ^a^	0.835	3270	3277	1.228	1659	1663
Monomer	1.010 (N15H)	3395	3395	1.222 (C17O)	1710	1710
Dimer	1.008 (N15H)	3433	3433	1.228 (C17O)	1688	1688
	1.014 (N46H)	3347	3347	1.226 (C48O)	1700	1700

a Ref [2].

#### 3.5.2 CH_2_ and NO_2_ vibration

Benznidazole have two CH_2_ groups. In the monomer, the asymmetric and symmetric vibration of the CH_2_ group was calculated at 3031, 2979 cm^−1^ and 2976, 2929 cm^−1^ respectively. Whereas, for the dimer asymmetric vibration was calculated at 3031, 2972 cm^−1^ and symmetric vibration was calculated to be 2959, 2920 cm^−1^. In the IR spectrum, the asymmetric stretching vibration of CH_2_ groups was observed at 3031, 2995 cm^−1^, and in the Raman spectra at 3058 and 2968 cm^−1^. The symmetric stretching vibration was detected in the IR spectra at 2966, and 2948 cm^−1^, and in the Raman spectra observed at 2956 cm^−1^. The scissoring and wagging were computed at 1464, 1455 cm^−1^, and 1370, 1355 cm^−1^ for the monomer, and the corresponding values for the dimer were calculated at 1484, 1465 cm^−1^ and 1372, 1333 cm^−1^. The infrared spectra scissoring and wagging were observed at 1454, 1467 cm^−1^, and 1357, 1368 cm^−1^, respectively. In the Raman spectra, they were displayed at 1471 cm^−1^ and 1361 cm^−1^. The imidazole ring contains a single NO_2_ group. For the monomer, the symmetric and asymmetric stretching vibrations of the NO2 group were computed at 1560 cm^−1^ and 1351 cm^−1^, and for the dimer, they were calculated to be 1554, 1542 cm^−1^, and 1366, 1369 cm^−1^. The infrared spectra of this group showed symmetric and asymmetric stretching vibrations of the nitro group observed at 1553 cm^−1^ and 1354 cm^−1^ in IR spectra. This observation led us to the conclusion that the experimental value and the computed vibrational modes for the CH_2_ group and NO_2_ group agree well.

#### 3.5.3 Ring R1 and R3 vibration

Ring R1 of monomer contains five CH moieties. In the dimer, the ring that corresponds to R1 is R3. The stretching vibrations of the CH moieties were calculated at 3049 and 3016 cm^−1^ in the monomer, 3050 and 3018 cm^−1^ in the ring R1, and 3048 and 3039 cm^−1^ in the ring R3 of the dimer. The stretching vibration of CH was observed at 3065, 3008 cm^−1^ in the IR spectra and 3069, 3014 cm^−1^ in the Raman spectra. The in-plane deformation of CH for the monomer was calculated to be 1503, 1346, and 1191 cm^−1^, for the dimer in ring R1 to be 1505, 1334, and 1194 cm^−1^, and for ring R3 to be 1504, 1342, and 1191 cm^−1^. In-plane deformation of CH was observed at 1502, 1348 cm^−1^ in IR spectra and 1546, 1183 cm^−1^ in Raman spectra. The out-of-plane deformation of CH was calculated at 1001, 987 cm^−1^ in monomer, 991, 934 cm^−1^ in ring R1, and 1002 and 988 cm^−1^ in R3 of dimer. The out-of-plane deformation of CH was observed at 993, 983 cm^−1^ in IR spectra, and 993 cm^−1^ in Raman spectra. The C-C stretching for monomer was calculated at 1326, 1099 cm^−1^, and in ring R1 of dimer it was 1100, 1041 cm^−1^; in ring R3 it was computed to be 1099, 1040 cm^−1^. The C-C stretching was observed at 1325, 1084 cm^−1^ in the IR spectra, and 1087 cm^−1^ in the Raman spectra. Based on this observation, we deduced that the calculated vibrational modes for rings R1 and R3 and the experimental value agree fairly well.

#### 3.5.4 Ring R2 and R4 vibration

The CH stretching vibrations were calculated at 3121, 3101 cm^−1^ for the monomer. In the dimer, ring R4 corresponds to ring R2 and the corresponding vibrations were calculated at 3116, 3100 cm^−1^ in ring R2, and 3116, 3101 cm^−1^ in ring R4 of the dimer. The stretching vibrations of the CH moieties within ring R2 were observed at 3129, 3113 cm^−1^ in the IR spectra and at 3133, 3117 cm^−1^ in the Raman spectra. In the monomer C−C stretching vibrations were computed to be 1500 cm^−1^ and, they were calculated to be 1503 cm^−1^ in ring R2 and 1501 cm^−1^ in R4 of the dimer. The C-C stretching vibration for ring R2 was observed at 1499 cm^−1^ in IR spectra and 1503 cm^−1^ in Raman spectra. The stretching vibrations of CN for the monomer were computed to be 1403 cm^−1^ and 1288 cm^−1^. In the dimer for ring R2, the vibrational wavenumbers were calculated at 1492 cm^−1^ and 1403 cm^−1^, and for ring R4, at 1493 and 1406 cm^−1^. In the Raman spectrum, the stretching vibration of CN in ring R2 was observed at 1395, 1291 cm^−1^, and 1414, 1289 cm^−1^ in the IR spectrum. This observation led us to the conclusion that the experimental value and the computed vibrational modes for the rings R2 and R4 agree well.

### 3.6 Molecular electrostatic potential (MEP) analysis

**Fig. 13:**
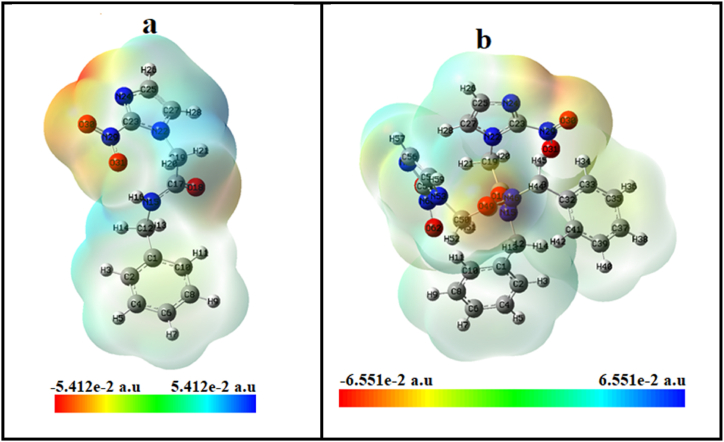
MEP map showing the distribution of electrostatic potential on the surface of (a) monomer, and (b) dimer of benznidazole.

MEP surface analysis is determined by the distribution of electron density and the configuration of atoms within the molecule. Reactive sites and the structural activity of biomolecules can be predicted based on MEP analysis [56]. The electrostatic potential on the molecule's surface is represented by different colors. The red color typically indicates regions with the highest electron density or the most negative electrostatic potential on the surface of a molecule. The areas with the highest positive electrostatic potential are represented by blue, and the areas with the zero potential are represented by green [57,58]. The MEP map for the monomer and dimer of benznidazole is presented in Fig. 13(a and b).

The highest negative electrostatic potential is shown by the oxygen atoms in the carbonyl group (C=O), oxygen of the nitro group (−NO_2_), and nitrogen of the imidazole ring. These regions are indicated by the red color, which has an electron-donating tendency and attacks the part with low electron density. A positive electrostatic potential is indicated by the blue areas on the map near certain atoms, such as the hydrogen atoms in the amide group and the hydrogen of CH moiety in the imidazole group, as well as the carbon atom in the carbonyl group. These regions are the center of the nucleophilic attack, with relatively low electron density in comparison to the surrounding areas. The hydrogen atom in amide functions as an electrophile, whereas oxygen of carbonyl functions as a nucleophile in the monomer of benznidazole. This results in the formation of a dimer by a strong intermolecular hydrogen bond (N−H…O). It was found that nucleophiles of dimer had lower electronegativity than monomers.

### 3.7 Natural bond orbital (NBO) analysis

NBO analysis is a useful tool for investigating how atoms connect within molecules and how these molecules interact with one another. This is an efficient method for investigating the flow of charges between atoms or the interaction of different bonds in complex molecular systems [59]. The interaction strength is based on the estimation of stabilization energy E(2) using second-order perturbation theory. Higher stabilization energy values denote stronger bonding or interaction between electron donors and acceptors by indicating a more potent interaction between them [60]. The interaction between donor and acceptor with their stabilization energies for the monomer and dimer of benznidazole was presented in Tables S7 and S8. With a maximum stabilization energy value of 126.90 kcal/mol, the monomer interaction LP(3)O31→ π∗(N29-O30) provides evidence for the presence of intra-molecular hydrogen bonds between H20 and O31. The molecular graph obtained from the QTAIM analysis provided more evidence for this. When it comes to monomer lone pair interactions, the stabilization energies for LP(1)N15→ π∗(C17-O18), LP(2)O18→ σ∗(N15-C17)/σ∗(C17-C19), and LP(1)N22→ π∗(C23-N24)/π∗(C25-C27) have the highest values (71.79, 22.97, 21.16, 46.96, and 33.13) kcal/mol respectively. The lone pair donors in the dimer of benznidazole to its acceptor anti-bonding are LP(1)N15→ π∗(C17-O18), LP(2)O18→ σ∗(N15-C17)/σ∗(C17-C19), LP(1)N22→π∗(C23-N24)/π∗(C25-C27), LP(3)O31→π∗(N29-O30), LP(1)N46→ π∗(C48-O49), and LP(2)O62→ π∗(N60-O62) have the largest stabilization energies that are (56.47, 21.55, 20.56, 46.98, 33.22, 139.97, 74.67, and 148.12) kcal/mol respectively. These interactions play a vital role in the stability of dimerized benznidazole. The maximum delocalization energy of 21.09 kcal/mol from π(C8-C10) → π∗(C1-C2) and σ (N24-C25) → σ∗(C23-N29) was 8.25 kcal/mol in dimer. Delocalization energy in monomer was found to be 21.01 kcal/mol for interactions from π(C8-C10) → π∗(C1-C2), and 8.00 kcal/mol for σ (N24-C25) → σ∗(C23-N29).

The percentage contribution of the s and p orbitals for the hybridization of atomic orbitals of atoms A and B, which participate in intermolecular hydrogen bonding and other interactions in both the monomer and dimer, is presented in Table S9. This data highlights the stabilizing effects of the interactions. The bonding orbitials π (C1-C2), π (C4-C6), and π (C8-C10) of monomer and dimer in the benzyl ring have sp hybridization, in which the contribution of s and p is about s(0.00 %) p(99.95 %). The σ(N15-H16) bonding orbital, which is a group of intermolecular hydrogen bonding, is formed by the hybridization of sp^2.46^ nitrogen with hydrogen s(99.91 %) in the monomer. This orbital was created in the dimer by sp^2.65^ hybridized nitrogen with hydrogen, which has a purely s contribution of s(99.92 %). In bond orbital σ(N15-H16), nitrogen is more electronegative in monomer and dimer with a higher value of polarization coefficient. In the monomer, the anti-bonding orbitals σ∗(C17-O18) and π∗(C17-O18) consist of s(0.02 %) and p(99.50 %) contributions in both cases. In the dimer, the contributions to σ∗(C17-O18) are s(30.37 %) and p(69.45 %) and for π∗, the contribution of s and p is s(2.39 %) and p(97.10 %). This variation in the dimer occurs due to intermolecular hydrogen bonding formed by the interaction LP(1)O18→ σ∗(N46-H47). The bond orbital LP(1)O18 is sp hybridized, which interacts with anti-bonding σ orbital formed by sp^2.40^ hybridized N46 with H47 having 99.91 % contribution of s.

### 3.8 Frontier molecular orbitals (FMO) analysis

**Fig. 14:**
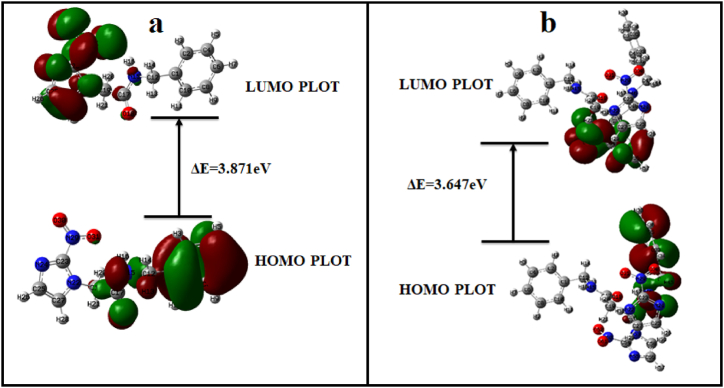
HOMO−LUMO plots for (a) monomer, and (b) dimer of benznidazole in gaseous state.

The highest occupied molecular orbital (HOMO) and lowest unoccupied molecular orbital (LUMO) generate the respective basic idea to study electron-donating capacity and electron-deficient capacity for chemical reactions. Nucleophiles often transfer electrons from their HOMO, whereas electrophiles often accept electrons into their LUMO during reactions [61,62]. Molecule stability and reactivity are explained by the energy gap (ΔE_L-H_). Smaller energy gap, indicates easier electron transfer, which increases a molecule's reactivity in specific kinds of chemical reactions and reduces its stability [63,64]. The present investigation examined the chemical behavior of benznidazole monomer and dimer in both gaseous and aqueous media. The HOMO−LUMO plots of monomer and dimer of benznidazole in gaseous state are shown in Fig. 14(a) and (b), respectively. The calculated values of ΔE_L-H_ for monomers are 3.872 eV (gas), 3.755 eV (ethanol), 3.754 eV (methanol), and 3.753 eV (water). Those values for dimer are 3.647 eV (gas), 3.758 eV (ethanol), 3.761 eV (methanol), and 3.766 eV (water). This observation leads to the conclusion that the dimer reactivity increases and stability decreases in the gaseous medium as compared to monomer, whereas calculations in solvent phases show that monomer is more reactive due to the ease of accessing charge transfer properties in a solvent.

The TD-DFT method was used in the IEF polarized continuum model in solvent water to study electronic transitions in monomers and dimers [65,66]. The UV–Vis spectra of the monomer and dimer in water are presented in Fig. S3 (supplementary material). The major transition having higher oscillator strength with the percentage contribution of molecular orbitals is shown in Table S10. The first excited states for monomer and dimer have excitation energies of 3.27 eV and 3.29 eV with absorption wavelengths *λ*_max_ of 378.88 nm and 377.21 nm, respectively, which have very small oscillator strengths. The optically active transition for monomer and dimer occurred at 3.99 eV and 3.57 eV with absorption wavelengths of 310.01 nm and 346.58 nm, respectively, and they have higher oscillator strengths. This active transition is in good agreement with the experimental value of the UV–Vis absorption spectrum, which has an excitation energy of 3.83 eV and an absorption wavelength of 324 nm in water [67].

**Fig. 15:**
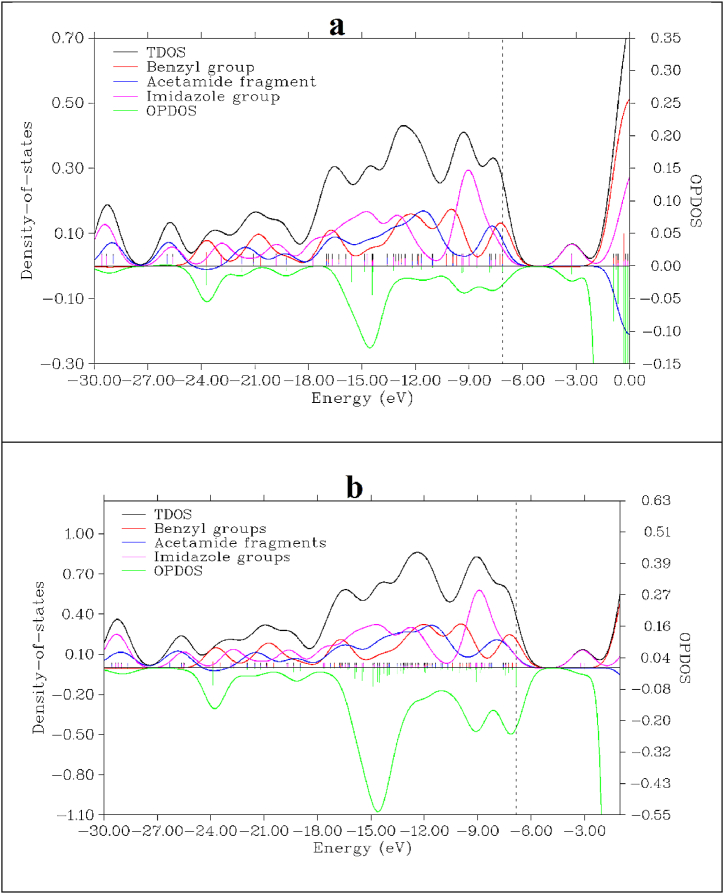
TDOS, PDOS and OPDOS of benznidazole for (a) monomer, and (b) dimer.

Plots of TDOS, PDOS, and OPDOS also visually represent the configuration of molecular orbitals (HOMO and LUMO) and their roles in the creation of chemical bonds. The composition of the fragment orbitals that contribute to the molecular orbitals is primarily displayed by the PDOS [68]. Positive regions in OPDOS denote energy levels dominantly occupied by bonding interactions, whereas negative regions denote energy levels dominated by anti-bonding interactions [69]. The visual representation of molecular orbital compositions for the monomer and dimer of benznidazole in terms of chemical bonding, TDOS, PDOS, and OPDOS population densities of states is shown in Fig. 15(a and b). The left side of the figure is the axis corresponding to the TDOS and PDOS, whereas the axis to the right corresponds to OPDOS. Since the acetamide and benzyl groups in the monomer and dimer exhibit poor overlap, the value of OPDOS negative in both cases indicates that the groups exhibit anti-bonding characteristics. The HOMO positions in the monomer and dimer, which have energies of −7.1139 eV and −6.8633 eV, respectively, are indicated by the vertical dashed line. Based on the TDOS graph, we observe that the molecule's benzyl ring contributes to HOMO and the acetamide group contributes to HOMO−1 at approximately −8.0000 eV in the monomer. Rather than HOMO, the imidazole group has contributed to LOMO. In the dimer, HOMO−1 behaves as an anti-bonding orbital between acetamide fragments and the benzyl group.

### 3.9 Global reactivity descriptors analysis

We evaluated different aspects of the molecule's reactivity at a quantum level by performing calculations involving FMO energies. Chemical reactivity descriptors, including electronegativity (χ), global softness (S), global hardness (η), chemical potential (μ), and electronegativity index (ω) are used to gain a better understanding of the compound's chemical reactivity. As suggested by Koopmans, in terms of HOMO and LUMO energy, the global reactivity descriptors can be evaluated [70]. A system that obtains the maximum amount of charge from its environment is indicated by the maximum charge transfer index ΔN_max_. The global reactivity descriptors of the benznidazole monomer and dimer for both the solvent and gaseous phases are shown in Table 3. When the benznidazole monomer and dimer were compared, the dimer was found to be more reactive than the monomer due to its greater softness and lower global hardness in the gaseous state. The softness and hardness of the monomer and dimer in the solvent phase were found to be nearly equal. The monomer has a higher electrophilicity index in the solvent phase, it behaves as a stronger electrophile in the solvent. Higher electronegativity monomers in both states are expected to be the best electron acceptors. The dimer exhibits greater chemical reactivity and is more polarizable than the monomer, with a smaller frontier gap in the gaseous medium.

**Table 3:** Calculated global reactivity descriptors for monomer and dimer of benznidazole gaseous phases and solvent phases.

Monomer									
Medium	E_H_(eV)	E_L_(eV)	(E_L_– E_H_)(eV)	χ (eV)	μ (eV)	η (eV)	S(eV^−1^)	ω (eV)	ΔNmax
Gaseous	−7.1139	−3.2420	3.8719	5.1779	−5.1779	1.9359	0.2582	6.9246	2.6746
Ethanol	−7.0595	−3.3048	3.7547	5.1821	−5.1821	1.8773	0.2663	7.1523	2.7604
Methanol	−7.0598	−3.3054	3.7544	5.1826	−5.1826	1.8772	0.2663	7.1392	2.7608
Water	−7.0598	−3.3068	3.7530	5.1833	−5.1833	1.8765	0.2664	7.1787	2.7622
**Dimer**									
Gaseous	−6.8633	−3.2161	3.6472	5.0397	−5.0397	1.8236	0.2741	6.9639	2.7636
Ethanol	−6.9866	−3.2281	3.7585	5.1013	−5.1013	1.8792	0.2660	6.9240	2.7146
Methanol	−6.9893	−3.2284	3.7609	5.1088	−5.1088	1.8804	0.2659	6.9399	2.7169
Water	−6.9947	−3.2287	3.7660	5.1115	−5.1115	1.8830	0.2655	6.9377	2.7145

E_H_ = Energy of HOMO, E_L_ = Energy of LUMO, E_L_– E_H_ = Energy gap, χ = Electronegativity, μ = Chemical potential, η = Global hardness, S = Global softness, ω = Global electrophilicity index, _ΔNmax=Charge transfer index._

### 3.10 Local reactivity descriptors analysis

Local reactivity descriptors are employed to recognize the reactivity of molecules at particular atomic locations. This is measured in terms of the Fukui function f(r), which identifies reactive sites in a molecule for nucleophilic and electrophilic attacks [71]. The sites in a molecule that are more reactive to electrophilic or nucleophilic attacks are predicted by the highest values of the FF at particular positions. The Fukui functions $f_{k}^{+}\left(r\right)$, $f_{k}^{-}\left(r\right)$,and $f_{k}^{0}\left(r\right)$, stands for nucleophilic, electrophilic and radical attack [34]. The local reactivity descriptors for monomer and dimer of benznidazole are presented in Tables S11 and S12. The Fukui calculations show that O30, O31, and N29 are the best electrophilic sites in the benznidazole monomer, while O18, N15, and C6 are the best nucleophilic sites. C17, C13, and H16 are the sites that are most vulnerable to radical attacks. The electrophilic sites in the dimer were identified as O62, O61, O30, N60, C58, and N29, while the nucleophilic sites were identified as C37, O49, C32, C6, C1, and C42. It was concluded that the sites C17, C48, C58, C23, C47, and N29 were susceptible to radical attacks.

### 3.11 Nonlinear optical (NLO) analysis

The NLO properties, which are not directly proportional to the intensity of the light, are used to study the interaction between light and matter. In our study, we study dipole moment, polarizability, anisotropy of polarizability, and first-order hyperpolarizability using the B3LYP/6−311++G(d,2p) level of theory. The dipole moment is crucial in understanding molecular structure as it indicates the charge movement resulting from electron donating and accepting groups. Polarizability and first-order hyperpolarizability measure how the dipole moment reacts to external electric fields [72]. The NLO materials have wide applications in the fields of optical data storage, biomedical imaging, laser technology, and telecommunications. Materials with strong nonlinear responses are necessary for the development of advanced optical devices and technologies. The mean polarizability $\left|\alpha_{0}\right|$ static dipole moment $\left(\mu_{0}\right)$, anisotropy of polarizability (Δα) and first-order hyperpolarizability $\left(\beta_{0}\right)$ defined in terms of *x, y,* and *z* coordinates used for their calculations [[73], [74], [75]]. Table 4 displays the benznidazole's static dipole moment, first-order hyperpolarizability, mean polarizability and anisotropy of polarizability. The higher contribution to dipole moment is due to μ_y_, with the highest negative value of −0.9111 Debye. This indicates that more electrons are located in the negative y-axis, potentially influencing molecular interaction and behavior. The total polarizability is due to the contributions of α_xx_, α_yy_, and α_zz_. The contribution of α_xx_ is higher in comparison to α_yy_ and α_zz_, indicating a higher response to the electric field along the x-axis. Urea is the standard molecule used to study NLO properties. The title molecule's total dipole moment and first-order hyperpolarizability were found to be 2.08 and 15.67 times larger than urea, respectively, indicating that it is a good candidate for NLO material [75].

**Table 4:** The anisotropy of polarizability (Δα), mean polarizability $\left|\alpha_{0}\right|$ dipole moment (μ_0_), and first hyperpolarizability (β_0_) of benznidazole calculated at B3LYP/6−311++G(d,2p) level.

Dipole moment^a^		Polarizability^b^		Hyperpolarizability^c^	
μ_x_	0.5566	α_xx_	227.7873	β_xxx_	−326.8790
μ_y_	−0.9111	α_xy_	13.6014	β_xxy_	−122.2188
μ_z_	−0.3549	α_yy_	200.0393	β_xyy_	137.5060
μ_0_	2.8600	α_xz_	−25.4185	β_yyy_	−514.3626
μ_0_(Urea)	1.3732	α_yz_	4.8353	β_xxz_	0.3455
		α_zz_	125.7632	β_xyz_	−44.5442
		$\left|\alpha_{0}\right|$	27.3473	β_yyz_	−163.7380
		Δα	60.1109	β_xzz_	8.2669
				β_yzz_	−3.8874
				β_zzz_	7.9587
				β_0_	5.8419
				β_0_(Urea)	0.3728

a Debye.

b = (∗10−^24^ esu), and.

c = (∗10−^30^ esu).

### 3.12 Thermodynamic properties

Thermal properties have been used to investigate the energy, structure, thermal stability, and decomposition of compounds at the molecular level [76]. Benznidazole has a thermal stability about to 274 °C, do not break down on melting to a limited temperature of 200 °C [77]. It melts at 192 °C, decomposes primarily between 234 and 320 °C, and remains thermally stable up to 234 °C [78]. The physical characteristics of a compound are clearly defined on the basis for calculating its thermodynamic properties, including free energy at different pressure and temperature levels [[79], [80], [81]]. This study used the B3LYP/6–311++G(d,2p) level of theory to calculate thermodynamic parameters, including specific heat capacity at constant pressure $\left(C_{p,m}^{0}\right)$, entropy $(S_{m}^{0}$), and enthalpy $\left(H_{m\;}^{0}\right)$, for benznidazole optimized in the temperature ranges from (50−850) K, which are listed in Table S13. The polynomial fit of the variation of thermodynamic functions with temperature and their regression values R^2^ are shown in Fig. S4. The R^2^ value should be nearer 1 for the best polynomial fit. The zero-point vibrational energy, total energy, entropy, and rotational constants are computed at room temperature (298.15 K) is shown in Table 5. The correlation equations for specific heat, enthalpy, and entropy of the benznidazole are given Fig. S4.

**Table 5:** The thermodynamic parameters for the monomer and dimer of benznidazole at 298.15 K using the B3LYP/6−311++G(d,2p) level of theory.

Parameters	Monomer	Dimer
Total energy (eV)	−24743.2524	−49486.9273
Specific heat (cal/mol-K)	60.550	126.912
Zero point energy (J/mol)	626208.1	1254680.6
Entropy (cal/mol-K)	138.575	236.973
Enthalpy (kcal/mol)	159.853	321.642
Rotational constant (GHz)	0.83931	0.09668

These equations can be used to determine the direction of a reaction and predict changes in its free energy; the influence of temperature on the properties of thermodynamics was predicted from the graph. The R^2^ value for enthalpy, specific heat capacity at constant pressure, and entropy was found to be close to 1 in the polynomial fit. This indicates that the thermodynamic parameters of the title compound are influenced by temperature changes, suggesting a correlation between them.

### 3.13 Drug likeness property

Swiss ADME web tools were used to measure several parameters of drug-likeness properties, such as molar refractivity, molecular weight, number of rotatable bonds, number of H-bond donors and acceptors, polar surface area, and AlogP [82,83]. To determine whether a compound has the potential to be developed into a safe and effective medication, these properties are crucial factors to take into account during the drug development process. The field widely acknowledges Lipinski's five rules, which offer a framework for the initial classification of drug candidates [84]. The drug-likeness parameters and their expected range given by Lipinski's five rules are presented in Table 6. This shows that benznidazole falls within the range of Lipinski's rules; hence, the drug is predicted to be non-toxic and can be accepted orally.

**Table 6:** Drug likeness parameters for the benznidazole.

Drug likeness parameters	Value	Range expected
Molecular weight	260.25 g/mol	<500
H-bond donors	1	<5
H-bond acceptors	4	<10
Number of rotatable bonds	6	<10
Polar surface area (PSA)	92.74 Å^2^	<140
AlogP	0.96	<5
Molar refractivity	69.41	40–130

### 3.14 Molecular docking

**Fig. 16:**
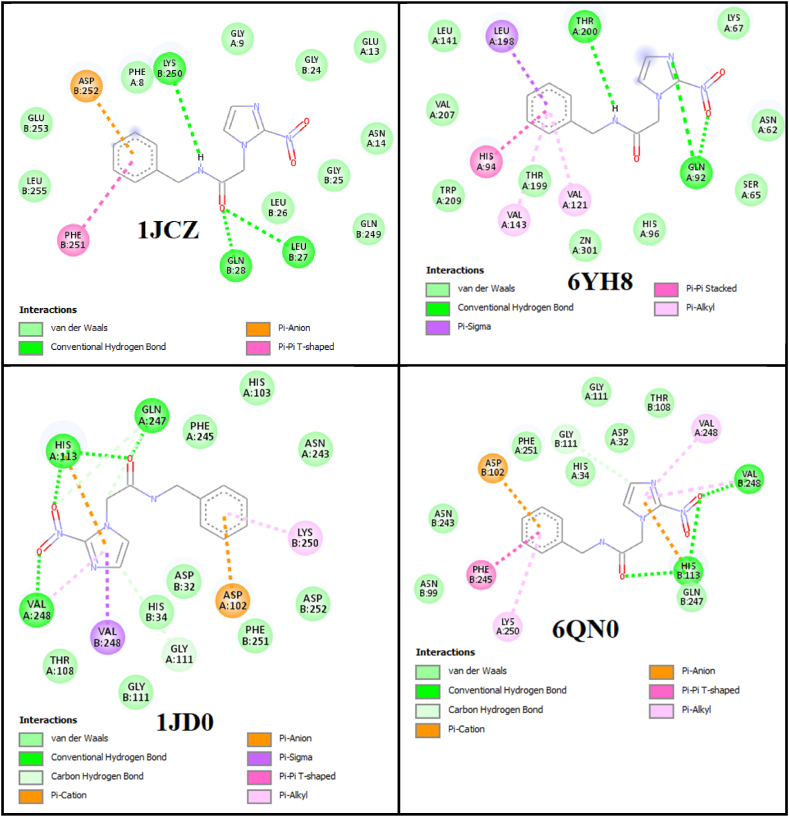
Docking of benznidazole with selected protein codes of carbonic anhydrase XII.

Molecular docking is an essential tool for computer-aided drug design. It predicts a drug's binding affinity with its targeted protein by identifying the most likely binding patterns. This prediction guides the effectiveness of the selected drug and evaluates its potential as a drug candidate [85]. The Swiss target prediction was utilized to predict the specific protein for the molecular docking of benznidazole [86,87]. The enzyme carbonic anhydrase XII was found to have a higher docking probability as predicted by Swiss Target Prediction shown in Fig. S5. The crystal structures of protein codes, which include the macromolecule protein carbonic anhydrase XII, was obtained from the RCSB data bank [88]. The protein codes 1JCZ, 1JD0, 6QN0, and 6YH8 contain macromolecule carbonic anhydrase XII, which was chosen and collected with their structures for molecular docking with benznidazole. The grid size has been selected as 60Å × 60Å × 60 Å with a spacing of 0.375 Å, to obtain the active sites of the protein. The binding modes and their interactions with the docked structure are presented in Fig. 16. The best binding mode and docking conformations of benznidazole, along with the hydrogen bond donor-acceptor regions of the protein, are shown in Fig. S6. The atoms involved in conventional hydrogen bonding, along with their corresponding bond lengths, the inhibition constant, and the binding affinity, ligand efficiency, and RMSD between the initial and docked structures are listed in Table 7.

**Table 7:** Molecular docking parameters of benznidazole with protein code (1JCZ, 1JD0, 6QN0, and 6YH8) of carbonic anhydrase XII.

Selected PDB code	Binding affinity (kcal/mol)	H-Bond residues	Atoms	Bond length(Å)	Ligand efficiency	Inhibition constant (μM)	RMSD (Å)
1JCZ	−7.7	GLN28	O18	1.97	0.41	2.23	1.92
		LYS250	H16	2.95			
		LEU27	O18	2.88			
1JD0	−8.0	GLN247	O18	2.28	0.35	1.35	1.41
		HIS113	O18	2.23			
		HIS113	O30	2.38			
		VAL248	O31	2.12			
6QN0	−8.2	VAL248	O30	2.11	0.43	0.96	1.37
		HIS113	O30	2.06			
		HIS113	O18	2.33			
6YH8	−8.3	THR200	H16	2.55	0.44	0.81	1.87
		GLN92	O30	2.34			
		GLN92	N24	2.93			

#### 3.14.1 Docking of benznidazole with 1JCZ

The cartesian coordinate in the grid box is taken as x = 4.442, y = −6.226, and z = 23.152, to obtain the active binding sites with the protein 1JCZ. The ligand demonstrated a strong binding affinity (−7.7 kcal/mol) along with a good inhibition constant (2.23 μM). The initial and docked structures had a very small RMSD, 1.92 Å. Hydrogen bond interactions were observed in the range (1.97−2.95) Å between the atoms O18 and H16 and residues GLN28, LEU27, and LYS250, respectively. Lower inhibition constant, higher negative binding affinity value, and minimal RMSD all highlight the molecule's effectiveness as an inhibitor for 1JCZ.

#### 3.14.2 Docking of benznidazole with 1JD0

The protein 1JD0 revealed active binding sites centered at coordinates x = 4.534, y = −6.398, and z = 23.219, which were pinpointed within a defined grid box. The ligand had a strong binding affinity of −8.0 kcal/mol and an impressive inhibition constant of 1.35 μM. The RMSD between the initial and docked structures was 1.41 Å. Notable hydrogen bonding interactions took place between bond lengths (2.12−2.38) Å, with atoms O18, O30, and O31 with residues GLN247, HIS113 and VAL248, respectively. When an acetazolamide inhibitor was docked with the carbonic anhydrase XII protein (code 1JD0), the docking result indicated a binding affinity of −6.71 kcal/mol [89]. This shows that benznidazole, an effective inhibitor of 1JD0 highlighted by its lower inhibition constant, higher negative binding affinity value, and minimal RMSD, all of which classify it as a potent inhibitor.

#### 3.14.3 Docking of benznidazole with 6QN0

The 6QN0 encoded protein displayed active binding sites that were located within a designated grid box at coordinates x = 4.524, y = −6.374, and z = 23.216. The ligand exhibited a lower inhibition constant of 0.96 μM along with a strong binding affinity of −8.2 kcal/mol. With an RMSD of 1.37 Å, the difference between the original and docked structures was very small. Hydrogen bonds occurred between bond lengths (2.06−2.33) Å, with atom O30 and O18 and residues VAL248 and HIS113, respectively. The compound's ability to inhibit 6QN0 is demonstrated by its reduced inhibition constant, increased negative binding affinity value, and low (RMSD), all of which combine to make it an effective inhibitor.

#### 3.14.4 Docking of benznidazole with 6YH8

Protein 6YH8 binding sites were predicted to be located in the grid box with coordinates x = 4.554, y = −6.378, and z = 23.214 as its center. With a higher binding affinity of −8.3 kcal/mol and a lower inhibition constant of 0.81 μM, benznidazole demonstrates greater potency when docked with 6YH8. Between the docked structure and the original structure, there was an RMSD of 1.87 Å. The hydrogen bonding between atoms H16, O30 and N24 and residues THR200 and GLN92 took place in the range of bond length (2.34−2.93) Å. Benznidazole is a highly effective inhibitor of protein 6YH8, as evidenced by its lowest inhibition constant and highest negative binding affinity value.

## 4 Conclusion

The dimer structure of benznidazole was created by using the intermolecular hydrogen bonding N−H…O from the most stable conformer. The interaction energy of hydrogen bonding in the formation of dimers with counterpoise correction was found to be −13.21 kcal/mol. The calculated results were in good agreement with the experimental results obtained from FT-Raman and FT-IR spectroscopy, except for the functional groups amide and carbonyl, which exhibited a red shift due to intermolecular hydrogen bonding that, appeared in the dimers and crystal packing. The spectroscopic results indicate that the dimer exhibits better alignment with experimental results compared to the monomer due to intermolecular hydrogen bonding. The interaction energy for H20 … O31 in the monomer and H47 … O18 in the dimer was large, according to the QTAIM analysis, indicating stronger hydrogen bonding. Nucleophiles of dimer had lower electronegativity observed on the molecular electrostatic potential surface. The maximum stabilization energy of 126.90 kcal/mol for the LP(3)O31→ π∗(N29-O30) interaction provides evidence for the presence of intra-molecular hydrogen bonding in the monomer. The optically active electronic transition for monomer and dimer in solvent water occurred at 310.01 nm and 346.58 nm, which was found to be closer to the experimental value of 324 nm. In comparison to the dimer, the monomer functions as a stronger electrophile in the solvent phase because of its higher electrophilicity index. The best nucleophilic sites of the dimer predicted by Fukui calculations were C37, O49, C32, C6, C1, and C42. The nucleophilic sites in the monomer were found to be O18, N15, and C6. The significant NLO properties predicted that the title compound could be used as an optical material. The temperature and the thermodynamic properties of the title compound were correlated. The molecular docking with carbonic anhydrase XII protein codes 1JCZ, 1JD0, 6QN0, and 6YH8 yields the highest negative binding affinity −7.7 kcal/mol to −8.3 kcal/mol with a low inhibition constant, indicating that benznidazole can inhibit carbonic anhydrase XII.

## CRediT authorship contribution statement

**Tirth Raj Paneru:** Writing – original draft, Methodology, Formal analysis, Conceptualization. **Manoj Kumar Chaudhary:** Writing – original draft, Methodology, Conceptualization. **Poonam Tandon:** Supervision, Software. **Bhawani Datt Joshi:** Writing – review & editing, Supervision, Formal analysis. **Beatriz Pinheiro Bezerra:** Visualization, Data curation. **Alejandro Pedro Ayala:** Validation, Data curation.

## Data availability statement

Data included in the article/supplementary material is referenced in the article.

## Ethics declarations

Review and/or approval by an ethics committee is not needed for this study because we don't work with humans or animals.

## Declaration of competing interest

The authors declare that they have no known competing financial interests or personal relationships that could have appeared to influence the work reported in this paper.
