# Supplementary material for: Spectroscopic (FT-IR and FT-Raman) and quantum chemical study on monomer and dimer of benznidazole from DFT and molecular docking approaches
Source: Heliyon. 2025 Jan 20;11(3):e42104. doi: 10.1016/j.heliyon.2025.e42104 (PMC11800084; doi:10.1016/j.heliyon.2025.e42104)
Supplement: Multimedia component 1 [file mmc1.docx]

**Supplementary Material**

**Spectroscopic (FT-IR and FT-Raman) and quantum chemical study on monomer and dimer of benznidazole from DFT and molecular docking approaches**

Tirth Raj Paneru^a,b^, Manoj Kumar Chaudhary^c^, Poonam Tandon^d*^, Bhawani Datt Joshi^e*^, Beatriz Pinheiro Bezerra^f^ , Alejandro Pedro Ayala^f^

*^a^Central Department of General Science, Far Western University, Mahendranagar, 10400, Nepal*

*^b^Central Department of Physics, Tribhuvan University, Kirtipur, Kathmandu, Nepal*

*^c^Department of Physics, Tribhuvan University, Amrit Campus, Institute of Science and Technology, Kathmandu 44600, Nepal*

*^d^Deen Dayal Upadhyaya Gorakhpur University and University of Lucknow, Lucknow-226007, India*

*^e^Department of Physics, Tribhuvan University, Siddhanath Science Campus, Mahendranagar, 10400, Nepal*

*^f^Department of Physics, Federal University of Ceará, Fortaleza CE 60440-900, Brazil*

**Corresponding authors:tandon_poonam@lkouniv.ac.in*, *https://orcid.org/0000-0002-8120-0498 (P. Tandon); bhawani.joshi@snsc.tu.edu.np, pbdjoshi@gmail.com, https://orcid.org/0000-0003-3276-9319 (B.D. Joshi, Phone:+977-9841580777, Fax:+977-99521304*

**FIGURES:**

| 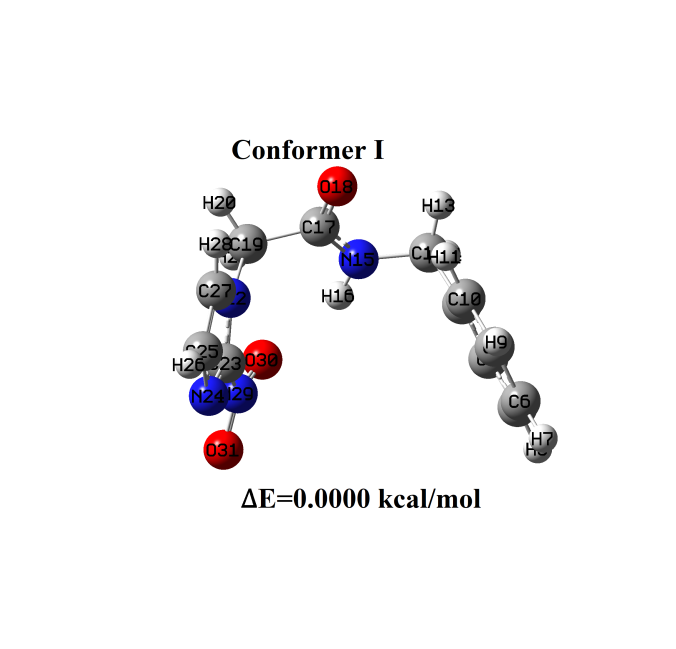 | 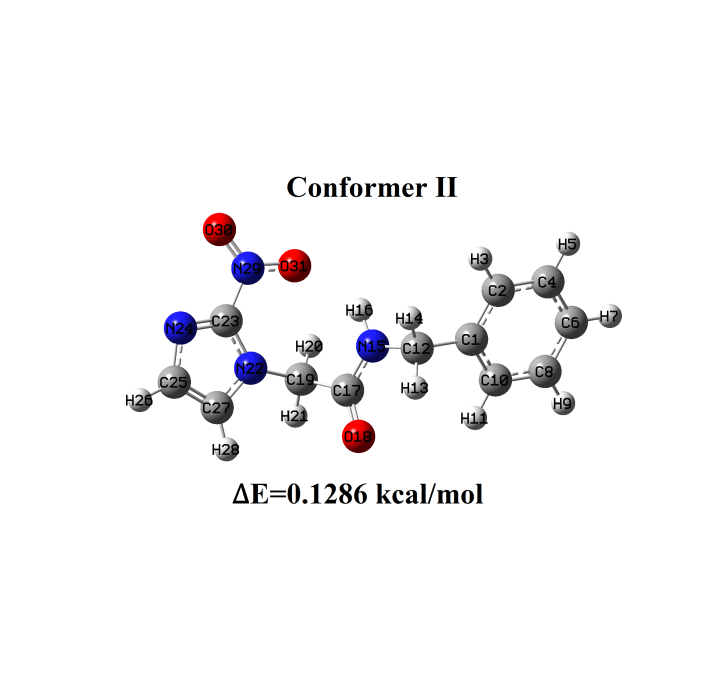 |
| --- | --- |
| 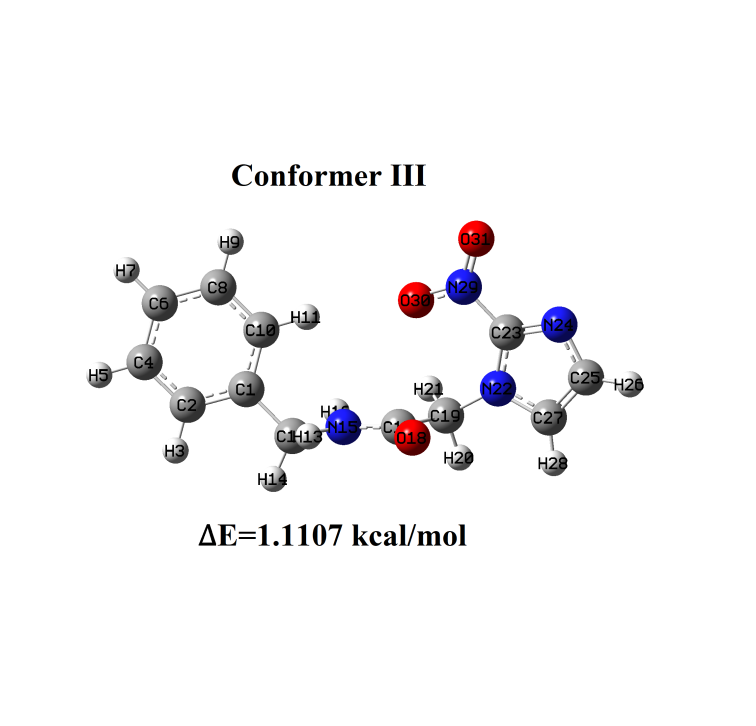 | 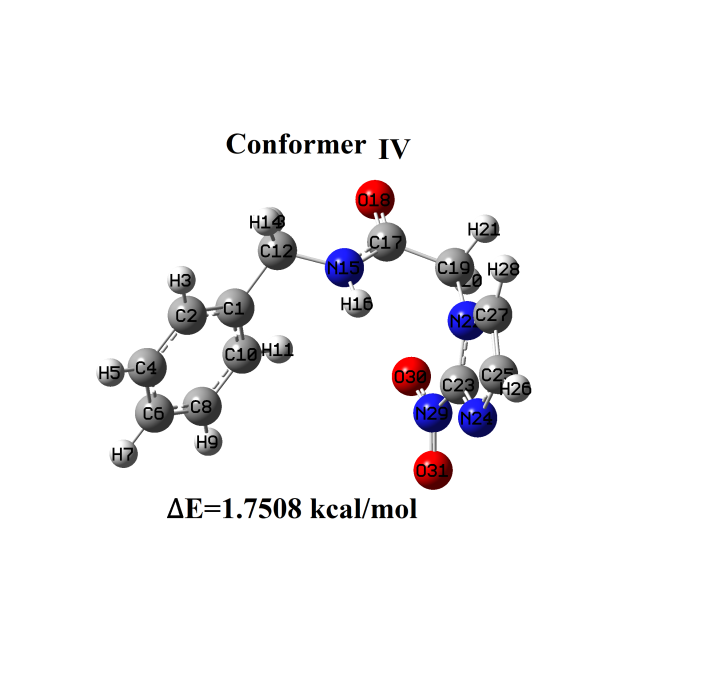 |
| 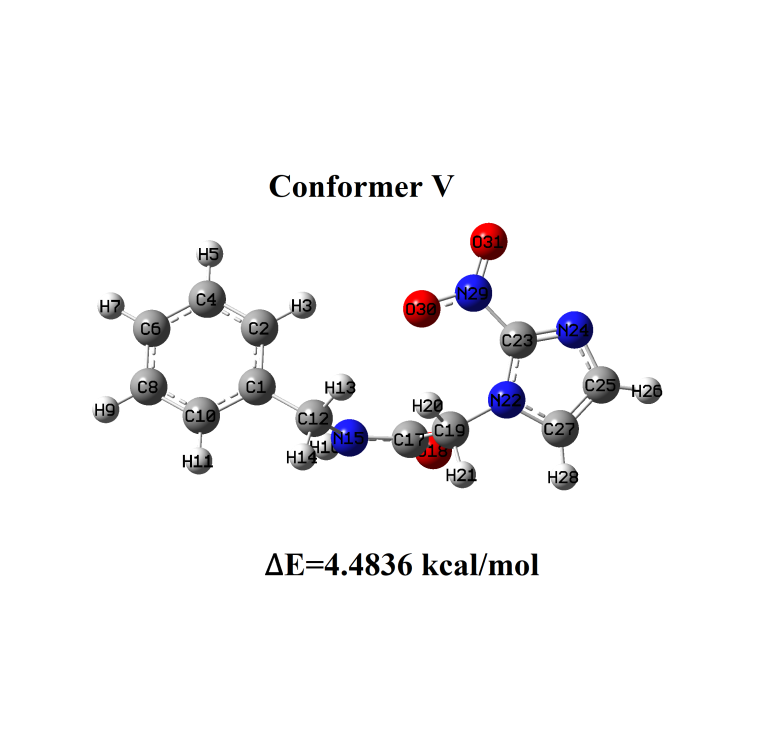 | |

**Fig. S1**. Optimized structure of five conformers of benznidazole at B3LYP/6−311++G(d,2p) level of theory with relative energies with respect to conformer I.

| 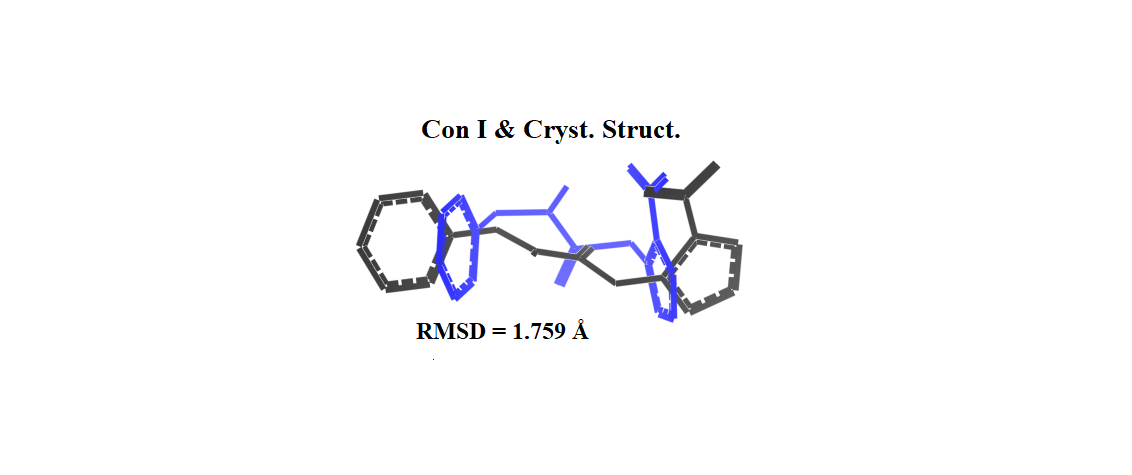 | 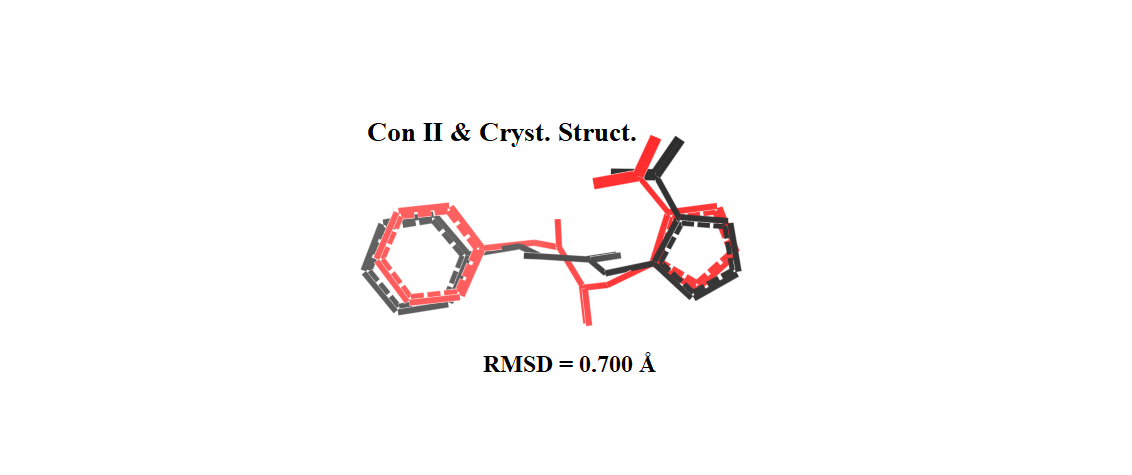 |
| --- | --- |
| 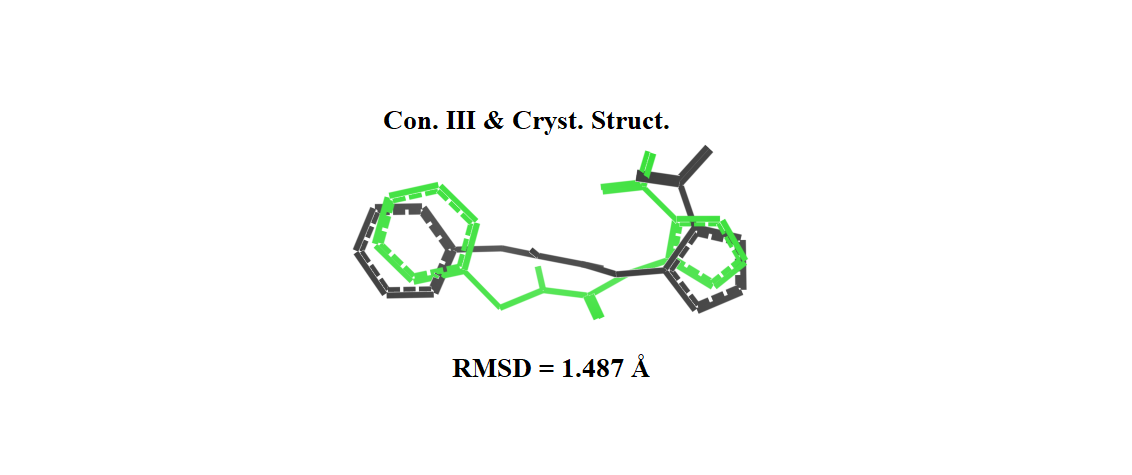 | 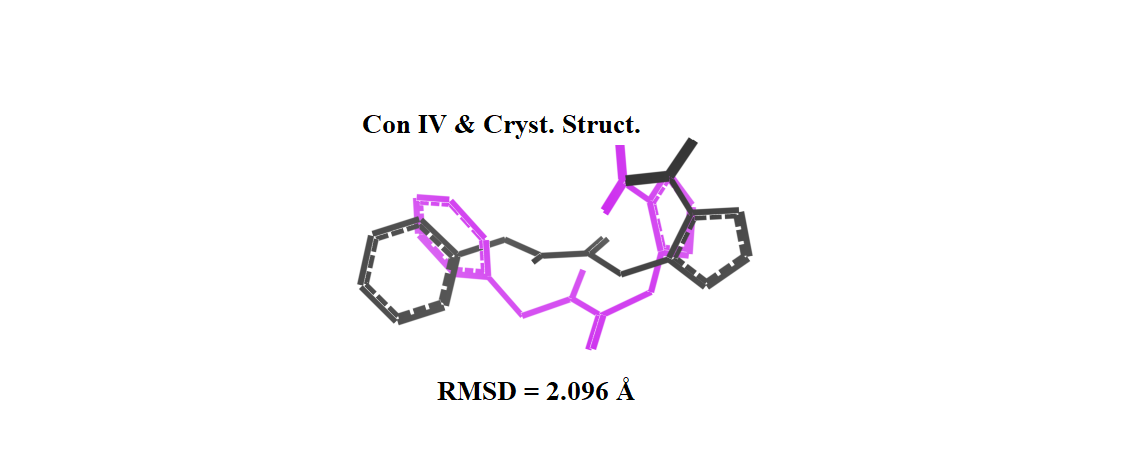 |
| 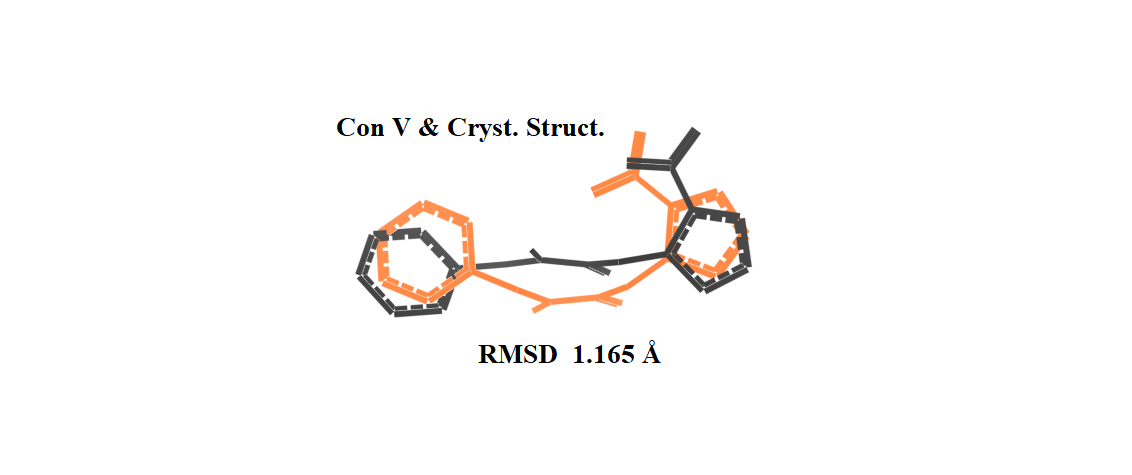 | |

**Fig. S2.** Overlapping between the crystal structure (black) and possible conformers (I–V) of benznidazole obtained by the B3LYP/6−311++G(d,2p) level of theory.


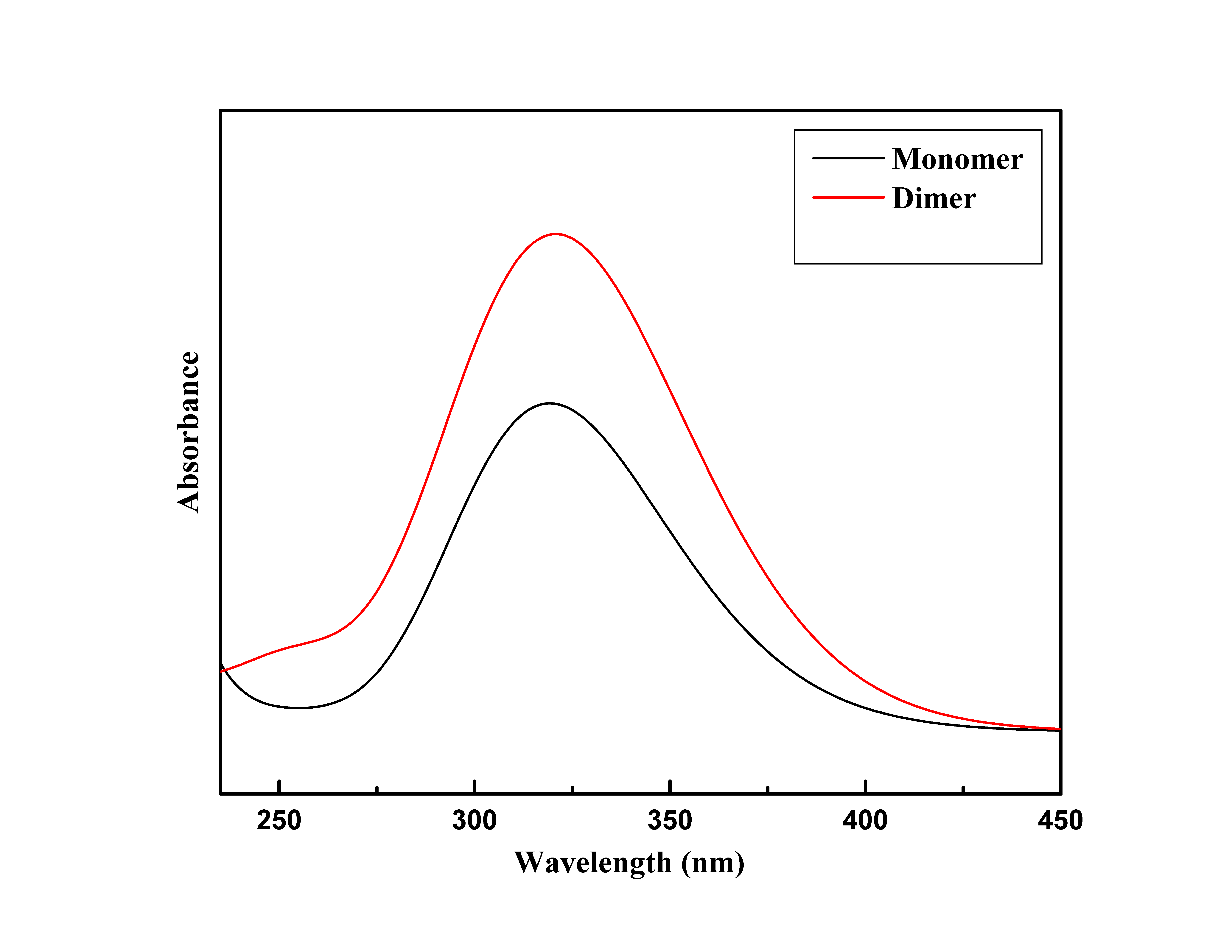


Fig. S3. The calculated UV−Vis absorption spectra for the monomer and dimer of benznidazole in solvent water.


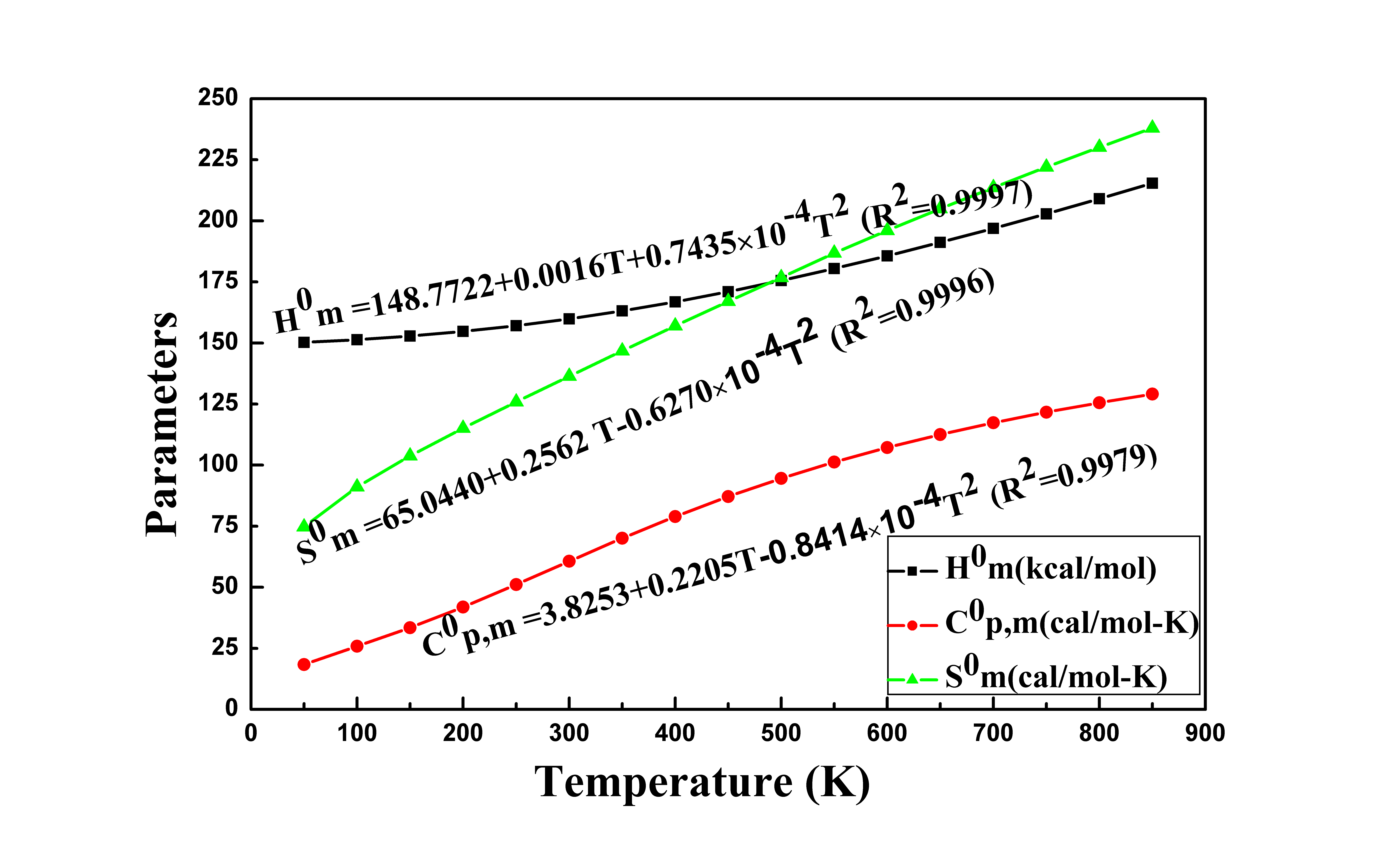


**Fig. S4.** Correlation graph with temperature for specific heat capacity$(C_{p,m}^{0})$, enthalpy$H_{m}^{0}$, and entropy$(S_{m}^{0}$) of benznidazole.


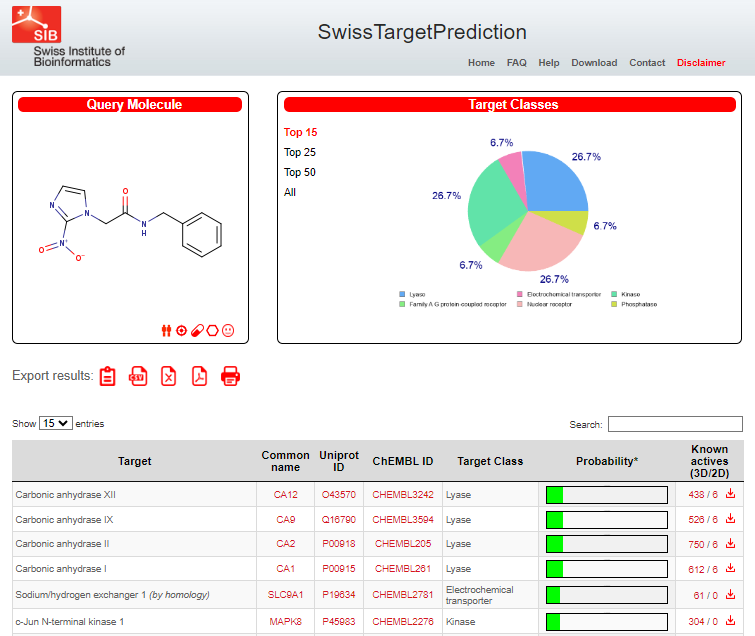


**Fig. S5.** Swiss target prediction for the prediction of protein.

| 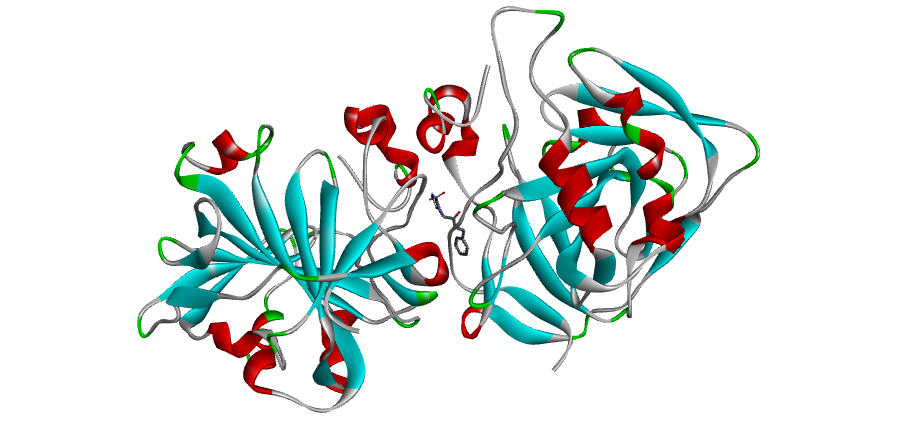  **1JCZ** | 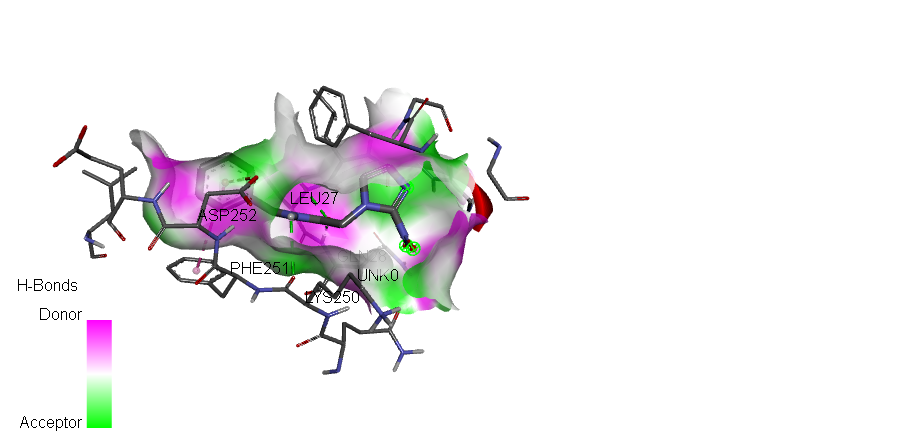  **1JCZ** |
| --- | --- |
| 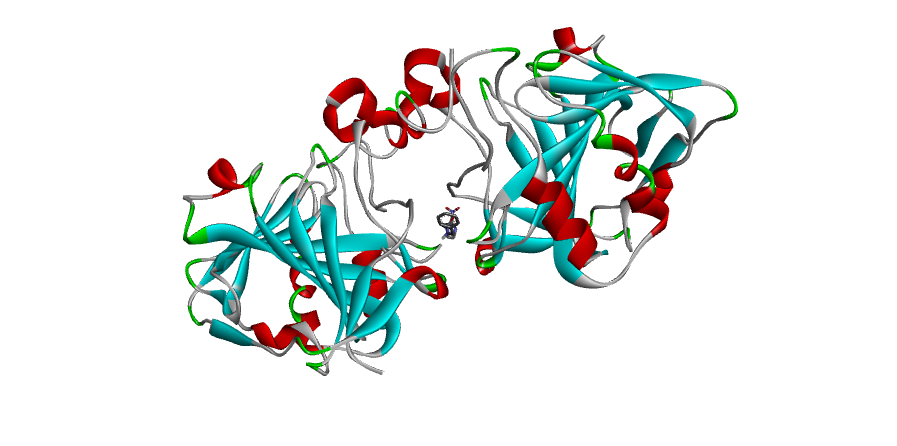  **1JD0** | 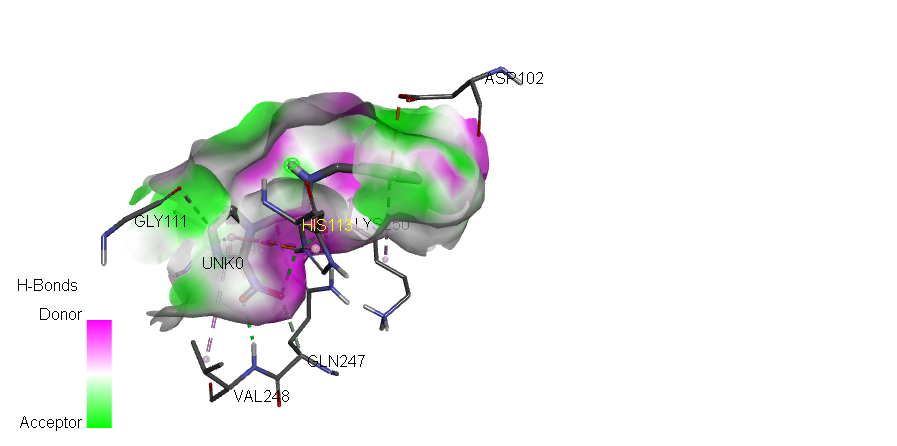  **1JD0** |
| 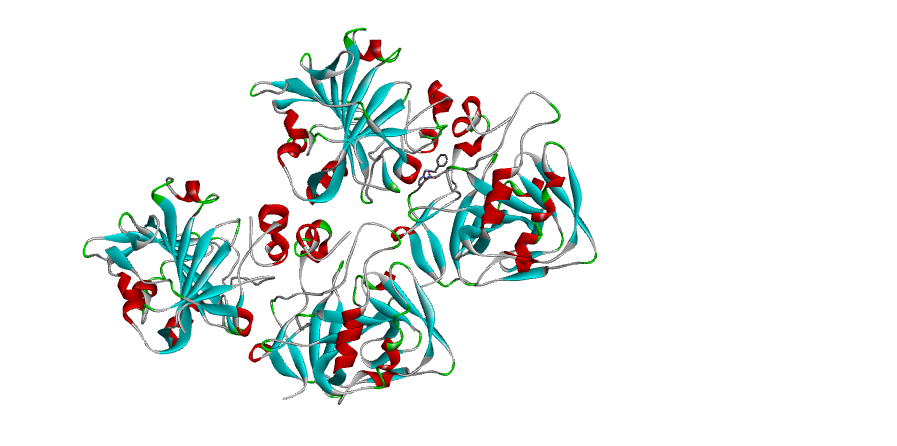**6QNO** | 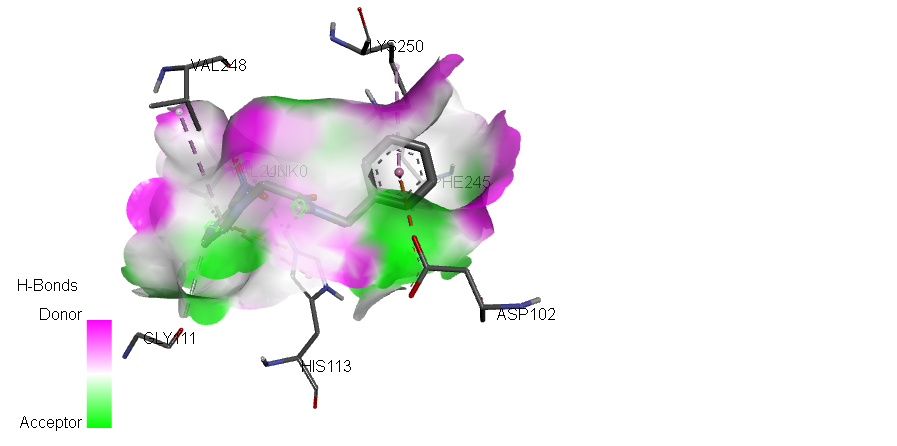**6QNO** |
| 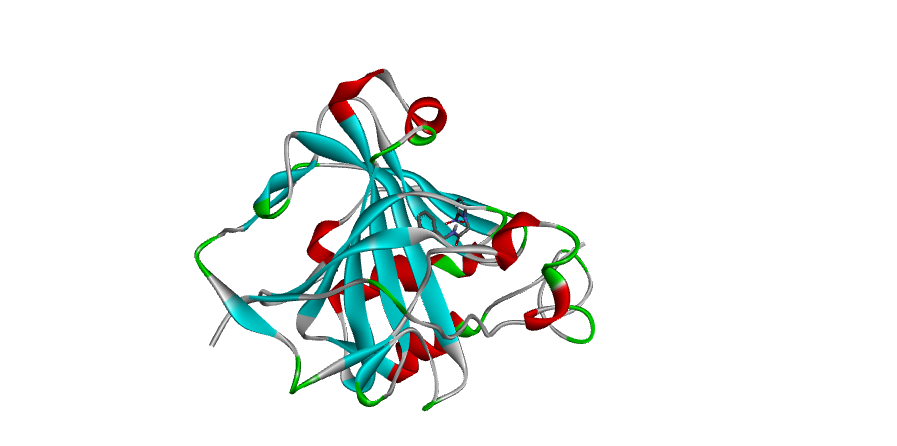  **6YH8** | 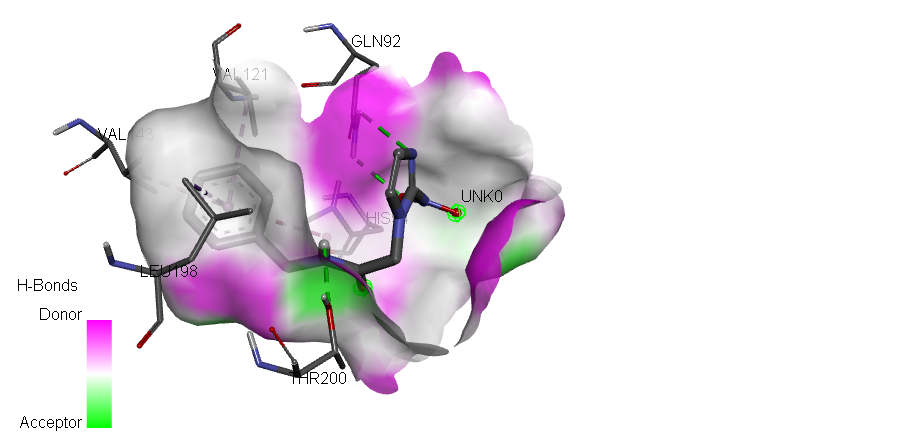  **6YH8** |

**Fig. S6.** Molecular docking conformations with best binding modes showing H-bond donor acceptor regions of protein codes 1JCZ, 1JD0, 6QN0, and 6YH8.

**TABLES:**

**Table S1.** Five possible conformers of benznidazole at room temperature, using B3LYP/6−311++G(d,p) and B3LYP/6−311++G(d,2p) level of theory.

| B3LYP/6−311++G(d,p) | | | | |
| --- | --- | --- | --- | --- |
| Conformer | **Dihedral Angle (°)** | **Energy**  **(Hartree)** | **Energy**  **(kcal/mol)** | **Energy Difference^*^**  **(kcal/mol)** |
| I | Φ_2_(C23-N22-C19-C17) | −909.339208 | −570618.9917 | 0 |
| II | Φ_1_(N24-C23-N29-O30) | −909.339029 | −570618.8794 | 0.1123 |
| III | Φ_2_(C23-N22-C19-C17) | −909.337490 | −570617.9137 | 1.0781 |
| IV | Φ_3_(N22-C19-C17-N15) | −909.336406 | −570617.2335 | 1.7583 |
| V | Φ_4_(C19-C17-N15-C12) | −909.332100 | −570614.5314 | 4.4603 |
| B3LYP/6−311++G(d,2p) | | | | |
| I | Φ_2_(C23-N22-C19-C17) | −909.344285 | −570622.1776 | 0 |
| II | Φ_1_(N24-C23-N29-O30) | −909.34408 | −570622.0490 | 0.1286 |
| III | Φ_2_(C23-N22-C19-C17) | −909.342515 | −570621.0669 | 1.1107 |
| IV | Φ_3_(N22-C19-C17-N15) | −909.341495 | −570620.4269 | 1.7508 |
| V | Φ_4_(C19-C17-N15-C12) | −909.33714 | −570617.6941 | 4.4836 |

^*Relative energies of five conformers with reference to minimum energy conformer I^

**Table S2.** The two most stable conformers of benznidazole, along with their energies, are shown using functional B3LYP and WB97XD with different basis sets.

| **Dihedral Angle** | **Conformer** | **B3LYP/6−311++G(d,p)** | | **WB97XD/6−311++G(d,p)** | |
| --- | --- | --- | --- | --- | --- |
|  |  | **Energy**  **(Hatree)** | **Energy**  **(kcal/mol)** | **Energy**  **(Hatree)** | **Energy**  **(kcal/mol)** |
| Φ_2_(C23-N22-C19-C17) | I | −909.339208 | −570618.9917 | −909.034259 | −570427.6333 |
| Φ_1_(N24-C23-N29-O30) | II | −909.339029 | −570618.8794 | −909.031047 | −570425.6179 |

| **Dihedral Angle** | **Conformer** | **B3LYP/6−311++G(d,2p)** | | **WB97XD/6−311++G(d,2p)** | |
| --- | --- | --- | --- | --- | --- |
|  |  | **Energy**  **(Hatree)** | **Energy**  **(kcal/mol)** | **Energy**  **(Hatree)** | **Energy**  **(kcal/mol)** |
| Φ_2_(C23-N22-C19-C17) | I | −909.344285 | −570622.1776 | −909.039089 | −570430.6642 |
| Φ_1_(N24-C23-N29-O30) | II | −909.34408 | −570622.049 | −909.035844 | −570428.6279 |

**Table S3.** The calculated (DFT) geometrical parameters (bond distance and bond angle) of benznidazole by using the functional B3LYP/6−311++G(d,2p) along with XRD structural parameters.

| Monomer | | Dimer | | | | XRD data^a^ |
| --- | --- | --- | --- | --- | --- | --- |
| Bonds | **Bond distance (Å)** | **Bonds** | **Bond distance (Å)** | **Bonds** | **Bond distance (Å)** | **Bond distance (Å)** |
| (C1-C2) | 1.397 | (C1-C2) | 1.397 | (C32-C33) | 1.397 | 1.388 |
| (C1-C10) | 1.400 | (C1-C10) | 1.401 | (C32-C41) | 1.400 | 1.395 |
| (C1-C12) | 1.516 | (C1-12) | 1.514 | (C32-C43) | 1.515 | 1.505 |
| (C2-H3) | 1.083 | (C2-H3) | 1.083 | (C33-H34) | 1.083 | 0.951 |
| (C2-C4) | 1.395 | (C2-C4) | 1.396 | (C33-C35) | 1.396 | 1.391 |
| (C4-H5) | 1.082 | (C4-H5) | 1.082 | (C35-H36) | 1.082 | 0.950 |
| (C4-C6) | 1.393 | (C4-C6) | 1.393 | (C35-C37) | 1.393 | 1.382 |
| (C6-H7) | 1.082 | (C6-H7) | 1.082 | (C37-H38) | 1.082 | 0.950 |
| (C6-C8) | 1.396 | (C6-C8) | 1.396 | (C37-C39) | 1.396 | 1.385 |
| (C8-H9) | 1.082 | (C8-H9) | 1.082 | (C39-H40) | 1.082 | 0.949 |
| (C8-C10) | 1.392 | (C8-C10) | 1.392 | (C39-C41) | 1.392 | 1.391 |
| (C10-H11) | 1.083 | (C10-H11) | 1.083 | (C41-H42) | 1.083 | 0.951 |
| (C12-H13) | 1.089 | (C12-H13) | 1.088 | (C43-H44) | 1.089 | 0.990 |
| (C12-H14) | 1.091 | (C12-H14) | 1.092 | (C43-H45) | 1.092 | 0.991 |
| (C12-N15) | 1.466 | (C12-N15) | 1.467 | (C43-N46) | 1.465 | 1.467 |
| (N15-H16) | 1.010 | (N15-H16) | 1.008 | (N46-H47) | 1.014 | 0.835 |
| (N15-C17) | 1.352 | (N15-C17) | 1.344 | (N46-C48) | 1.349 | 1.329 |
| (C17-O18) | 1.222 | (C17-O18) | 1.228 | (C48-O49) | 1.226 | 1.228 |
| (C17-C19) | 1.543 | (C17-C19) | 1.537 | (C48-C50) | 1.539 | 1.528 |
| (C19-H20) | 1.084 | (C19-H20) | 1.086 | (C50-H51) | 1.082 | 0.991 |
| (C19-H21) | 1.086 | (C19-H21) | 1.089 | (C50-H52) | 1.088 | 0.989 |
| (C19-N22) | 1.471 | (C19-N22) | 1.458 | (C50-N53) | 1.476 | 1.462 |
| (N22-C23) | 1.378 | (N22-C23) | 1.376 | (N53-C54) | 1.372 | 1.363 |
| (N22-C27) | 1.369 | (N22-C27) | 1.370 | (N53-C58) | 1.370 | 1.359 |
| (C23-N24) | 1.312 | (C23-N24) | 1.311 | (C54-N55) | 1.314 | 1.315 |
| (C23-N29) | 1.442 | (C23-N29) | 1.441 | (C54-N60) | 1.440 | 1.434 |
| (N24-C25) | 1.358 | (N24-C25) | 1.360 | (N55-C56) | 1.360 | 1.362 |
| (C25-H26) | 1.076 | (C25-H26) | 1.076 | (C56-H57) | 1.076 | 0.949 |
| (C25-C27) | 1.381 | (C25-C27) | 1.381 | (C56-C58) | 1.381 | 1.370 |
| (C27-H28) | 1.075 | (C27-H28) | 1.076 | (C58-H59) | 1.075 | 0.949 |
| (N29-O30) | 1.214 | (N29-O30) | 1.219 | (N60-O61) | 1.227 | 1.226 |
| (N29-O31) | 1.243 | (N29-O31) | 1.238 | (N60-O62) | 1.232 | 1.232 |
| - | - | (O18-H47) | 1.964 | - | - | - |
| RMSD | **0.077 (Å)** |  | **0.077 (Å)** |  | **0.077 (Å)** |  |
| Monomer | | **Dimer** | | | | **XRD Data^a^** |
| Bond Angle | **Angle ( ͦ )** | **Bond Angle** | **Angle ( ͦ )** | **Bond Angle** | **Angle ( ͦ )** | **Angle ( ͦ )** |
| (C2-C1-C10) | 119.0 | (C2-C1-C10) | 119.1 | (C33-C32-C41) | 118.9 | 118.9 |
| (C2-C1-C12) | 120.7 | (C2-C1-C12) | 120.5 | (C33-C32-C43) | 120.7 | 120.4 |
| (C10-C1-C12) | 120.3 | (C10-C1-C12) | 120.4 | (C41-C32-C43) | 120.4 | 120.7 |
| (C1-C2-H3) | 119.7 | (C1-C2-H3) | 119.6 | (C32-C33-H34) | 119.9 | 119.6 |
| (C1-C2-C4) | 120.7 | (C1-C2-C4) | 120.6 | (C32-C33-C35) | 120.6 | 120.8 |
| (H3-C2-C4) | 119.7 | (H3-C2-C4) | 119.8 | (H34-C33-C35) | 119.5 | 119.6 |
| (C2-C4-H5) | 119.9 | (C2-C4-H5) | 119.9 | (C33-C35-H36) | 119.6 | 120.1 |
| (C2-C4-C6) | 120.0 | (C2-C4-C6) | 120.0 | (C33-C35-C37) | 120.1 | 119.7 |
| (H5-C4-C6) | 120.2 | (H5-C4-C6) | 120.2 | (H36-C35-C37) | 120.3 | 120.2 |
| (C4-C6-H7) | 120.1 | (C4-C6-H7) | 120.1 | (C35-C37-H38) | 120.2 | 119.9 |
| (C4-C6-C8) | 119.7 | (C4-C6-C8) | 119.8 | (C35-C37-C39) | 119.7 | 120.3 |
| (H7-C6-C8) | 120.1 | (H7-C6-C8) | 120.1 | (H38-C37-C39) | 120.1 | 119.8 |
| (C6-C8-H9) | 120.0 | (C6-C8-H9) | 120.0 | (C37-C39-H40) | 120.0 | 120.1 |
| (C6-C8-C10) | 120.2 | (C6-C8-C10) | 120.3 | (C37-C39-C41) | 120.1 | 119.8 |
| (H9-C8-C10) | 119.8 | (H9-C8-C10) | 119.7 | (H40-C39-C41) | 119.8 | 120.1 |
| (C1-C10-C8) | 120.4 | (C1-C10-C8) | 120.3 | (C32-C41-C39) | 120.6 | 120.4 |
| (C1-C10-H11) | 119.5 | (C1-C10-H11) | 119.8 | (C32-C41-H42) | 119.6 | 119.8 |
| (C8-C10-H11) | 120.1 | (C8-C10-H11) | 119.9 | (C39-C41-H42) | 119.9 | 119.8 |
| (C1-C12-H13) | 110.6 | (C1-C12-H13) | 111.3 | (C32-C43-H44) | 111.1 | 109.5 |
| (C1-C12-H14) | 110.1 | (C1-C12-H14) | 110.4 | (C32-C43-H45) | 109.9 | 109.4 |
| (C1-C12-N15) | 113.1 | (C1-C12-N15) | 111.8 | (C32-C43-N46) | 112.2 | 110.9 |
| (H13-C12-H14) | 108.1 | (H13-C12-H14) | 107.5 | (H44-C43-H45) | 107.7 | 108.1 |
| (H13-C12-N15) | 106.6 | (H13-C12-N15) | 106.4 | (H44-C43-N46) | 106.5 | 109.5 |
| (H14-C12-N15) | 108.1 | (H14-C12-N15) | 109.4 | (H45-C43-N46) | 109.3 | 109.5 |
| (C12-N15-H16) | 119.0 | (C12-N15-H16) | 117.4 | (C43-N46-H47) | 118.0 | 119.8 |
| (C12-N15-C17) | 123.0 | (C12-N15-C17) | 123.9 | (C43-N46-C48) | 122.9 | 121.1 |
| (H16-N15-C17) | 118.0 | (H16-N15-C17) | 118.6 | (H47-N46-C48) | 118.8 | 118.3 |
| (N15-C17-O18) | 125.1 | (N15-C17-O18) | 124.3 | (N46-C48-O49) | 125.3 | 124.5 |
| (N15-C17-C19) | 114.9 | (N15-C17-C19) | 114.5 | (N46-C48-C50) | 114.6 | 114.5 |
| (O18-C17-C19) | 120.0 | (O18-C17-C19) | 121.0 | (O49-C48-C50) | 120.1 | 120.9 |
| (C17-C19-H20) | 112.2 | (C17-C19-H20) | 111.2 | (C48-C50-H51) | 112.5 | 109.4 |
| (C17-C19-H21) | 107.0 | (C17-C19-H21) | 107.6 | (C48-C50-H52) | 108.1 | 109.4 |
| (C17-C19-N22) | 111.5 | (C17-C19-N22) | 111.6 | (C48-C50-N53) | 110.1 | 110.8 |
| (H20-C19-H21) | 109.8 | (H20-C19-H21) | 109.4 | (H51-C50-H52) | 109.3 | 108.1 |
| (H20-C19-N22) | 108.9 | (H20-C19-N22) | 109.9 | (H51-C50-N53) | 108.0 | 109.5 |
| (H21-C19-N22) | 107.3 | (H21-C19-N22) | 107.0 | (H52-C50-N53) | 108.8 | 109.5 |
| (C19-N22-C23) | 131.8 | (C19-N22-C23) | 130.3 | (C50-N53-C54) | 129.7 | 130.7 |
| (C19-N22-C27) | 123.3 | (C19-N22-C27) | 124.4 | (C50-N53-C58) | 124.8 | 124.2 |
| (C23-N22-C27) | 104.9 | (C23-N22-C27) | 105.1 | (C54-N53-C58) | 105.0 | 105.0 |
| (N22-C23-N24) | 113.1 | (N22-C23-N24) | 113.1 | (N53-C54-N55) | 113.4 | 113.8 |
| (N22-C23-N29) | 124.3 | (N22-C23-N29) | 123.4 | (N53-C54-N60) | 123.9 | 123.4 |
| (N24-C23-N29) | 122.6 | (N24-C23-N29) | 123.5 | (N55-C54-N60) | 122.6 | 122.8 |
| (C23-N24-C25) | 105.1 | (C23-N24-C25) | 104.9 | (C54-N55-C56) | 104.6 | 103.8 |
| (N24-C25-H26) | 121.7 | (N24-C25-H26) | 121.7 | (N55-C56-H57) | 121.6 | 124.7 |
| (N24-C25-C27) | 110.3 | (N24-C25-C27) | 110.4 | (N55-C56-C58) | 110.5 | 110.7 |
| (H26-C25-C27) | 128.0 | (H26-C25-C27) | 127.9 | (H57-C56-C58) | 127.9 | 124.6 |
| (N22-C27-C25) | 106.7 | (N22-C27-C25) | 106.4 | (N53-C58-C56) | 106.4 | 106.8 |
| (N22-C27-H28) | 121.0 | (N22-C27-H28) | 121.4 | (N53-C58-H59) | 120.8 | 126.6 |
| (C25-C27-H28) | 132.3 | (C25-C27-H28) | 132.1 | (C56-C58-H59) | 132.7 | 126.6 |
| (C23-N29-O30) | 118.1 | (C23-N29-O30) | 118.1 | (C54-N60-O61) | 117.6 | 118.3 |
| (C23-N29-O31) | 117.4 | (C23-N29-O31) | 117.0 | (C54-N60-O62) | 117.9 | 117.6 |
| (O30-N29-O31) | 124.5 | (O30-N29-O31) | 124.9 | (O61-N60-O62) | 124.5 | 124.07 |
| - | - | (C17-O18-H47) | 162.6 | - | - | - |
| RMSD | **1.63˚** |  | **1.59˚** |  | **1.61˚** |  |

^a^[Ref. 2]

**Table S4.** The intra- and inter molecular interactions in monomer and dimer of benznidazole with the hydrogen bond geometry from DFT and XRD structure.

| **Hydrogen bond** | **Optimized structure (DFT)** | | | | **XRD structure (Crystal Structure)^a^** | | | |
| --- | --- | --- | --- | --- | --- | --- | --- | --- |
| D−H...A | D−H (A$\boldsymbol{^{\circ}}$) | H…A (A$\boldsymbol{^{\circ}}$) | D…A (A$\boldsymbol{^{\circ}}$) | D−H...A ($\boldsymbol{^{\circ}}$) | D−H (A$\boldsymbol{^{\circ}}$) | H...A (A$\boldsymbol{^{\circ}}$) | D...A (A$\boldsymbol{^{\circ}}$) | D−H...A ($\boldsymbol{^{\circ}}$) |
| Monomer (Intra-molecular hydrogen bonds) | | | | | | | | |
| N15 -H16...O31 | 1.010 | 2.103 | 2.982 | 145.2 | 0.834 | 5.660 | 5.586 | 80.7 |
| C19-H20...O31 | 1.084 | 2.285 | 2.856 | 110.8 | 0.990 | 4.121 | 4.322 | 95.6 |
| C10-H11...O18 | 1.082 | 2.966 | 3.699 | 125.3 | 0.950 | 4.590 | 4.899 | 103.5 |
| Dimer (Intermolecular hydrogen bonds) | | | | | | | | |
| N46-H47...O18 | 1.014 | 1.964 | 2.963 | 167.9 | 0.835 | 2.037 | 2.837 | 160.2 |

^a^ [Reference 2]

**Table S5**. Potential energy distribution of calculated and experimental wavenumbers (cm^-1^) for monomer of benznidazole.

| **Unscaled DFT** | **Scaled** | **IR** | **Raman** | **PED^a^(%)** |
| --- | --- | --- | --- | --- |
| 3572 | 3395 | 3270 | 3277 | ν(NH)(100) |
| 3266 | 3121 | 3129 | 3133 | R2[ν(CH)](99) |
| 3244 | 3101 | 3113 | 3117 | R2[ν(CH)](99) |
| 3187 | 3049 | 3065 | 3069 | R1[ν(CH)](100) |
| 3176 | 3039 | 3034 | - | R1[ν(CH)](99) |
| 3167 | 3032 | 3033 | 3061 | R1[ν(CH)](95) |
| 3167 | 3031 | 3031 | 3059 | ν_a_(C19H_2_)(96) |
| 3158 | 3023 | - | 3039 | R1[ν(CH)](99) |
| 3150 | 3016 | 3008 | 3014 | R1[ν(CH)](99) |
| 3110 | 2979 | 2995 | 2968 | ν_a_(C12H_2_) (99) |
| 3106 | 2976 | 2966 | 2956 | ν_s_(C19H_2_)(100) |
| 3054 | 2929 | 2948 | - | ν_s_(C12H_2_) (100) |
| 1743 | 1710 | 1659 | 1663 | ν(C=O)(73)+ν(C17N)(11)+δ(C=O)(7) |
| 1644 | 1614 | 1610 | 1611 | R1[ν(CC)](64)+R1[δ_in_(CH)](22)+R1[δ_a_](10) |
| 1625 | 1596 | 1586 | 1591 | R1[ν(CC)](68)+R1[δ_in_(CH)](18)+R1[δ_a_](9) |
| 1588 | 1560 | 1553 | - | ν_a_(NO_2_)(67)+ρ_in_(NH)(8)+δ_in_(NO2)(7)+R2[δ_in_ (C23N)](6) |
| 1568 | 1541 | 1535 | 1540 | ρ_in_(NH)(45)+ν(C17N)(19)+ν_s_(NO_2_)(15) |
| 1527 | 1503 | 1502 | 1506 | R1[δ_in_(CH)](59)+R1[ν(CC)](33) |
| 1524 | 1500 | 1499 | 1503 | R2[ν(CC)](60)+R2[δ_in_(CH)](25) |
| 1510 | 1486 | 1482 | 1485 | R2[ν(CN)](48)+δ(C19H_2_)(16)+R2[δ_in_(CH)](14)+ν_a_(NO_2_)(10)  +R2[δ_in_(C23N)](5) |
| 1487 | 1464 | 1467 | 1471 | δ(C12H_2_)(90) |
| 1485 | 1463 | 1463 | 1467 | R1[δ_in_(CH)](53)+R1[ν(CC)] (32)+[δ+γ+ρ](C12H_2_)(7) |
| 1477 | 1455 | 1454 | - | δ(C19H_2_)(69)+R2[ν(CC)](13)+R2[δ_in_(CH)](6) |
| 1424 | 1403 | 1414 | 1395 | R2[ν(CN)](62)+[γ+ρ] (C19H_2_)(15)+R2[δ_in_(CH)](7)+R2[δ_ring_](6) |
| 1390 | 1370 | 1368 | 1361 | ω(C12H_2_)(78)+R1[ν(C12C)](6) |
| 1374 | 1355 | 1357 | 1360 | ω(C19H_2_)(58)+R2[ν(CC)](14)+R2[ν(N29C)](7) |
| 1370 | 1351 | 1354 | 1342 | ν_s_(NO_2_)(37)+R2[ν(CC)](25)+[γ+δ](C19H_2_)(10)+δ(NO_2_)(9)  +R2[ν(N29C)](5) |
| 1364 | 1346 | 1348 | - | R1[δin(CH)](64)+[γ+ω](C12H_2_)(10)+R2[ν(N29C)](5 |
| 1360 | 1342 | 1343 | - | [ω+γ](C19H_2_)(26)+R2[ν(CC)](24)+R2[ν(N29C)](15)+R2[δin(CH)](7)+R2[δring](7)+R2[ν(C19N)](5) |
| 1344 | 1326 | 1325 | - | R1[ν(CC)](74)+R1[δ_in_(CH)](14)+γ(C12H_2_)(9) |
| 1304 | 1288 | 1289 | 1291 | R2[ν(CN)](23)+R2[δ_in_(CH)](20)+R2[ν(C19N)](16)+ν_s_(NO_2_)(13)+[γ+ω](C19H_2_)(11)+R2[δ_ring_](6) |
| 1290 | 1274 | 1277 | - | ν(C17N)(24)+ρ_in_(NH)(14)+[ρ_in_+ν](C=O)(13)+[ρ+γ+ω]+(C19H_2_)(11)+ν(CC)(9)+R2[ν(CC)](9) |
| 1271 | 1256 | 1247 | 1257 | γ(C12H_2_)(45)+R1[ν(CC)](31)+ρ_in_(NH)(8) ρ(C12H_2_)(6)+R1[δ_in_(CH)](6) |
| 1224 | 1210 | 1217 | 1217 | R1[ν(C12C)](37)+R1[ν(CC)](18)+R1[δ_in_(CH](13)+R1[δ_trig_](12) |
| 1219 | 1205 | 1211 | - | R2[ν(CC)](20)+γ(C19H_2_)(15)+R2[δ_in_(CH)](12)+R2[ν(C19N)](9)  +ν_s_(NO_2_)(9)+R2[δ_ring_](9)+R1[ν(C12C)](6) |
| 1204 | 1191 | - | 1183 | R1[δ_in_(CH)](78)+R1[ν(CC)](21) |
| 1191 | 1178 | 1187 | 1178 | R2[ν(CC)](33)+R2[δ_ring_](16)+R2[δ_in_(CH)](16)+R2[ν(CC)](9)  +R2[ν(N29C)](7)+γ(C19H_2_)(6)+R2[δ_in_(C23N)](5) |
| 1182 | 1170 | 1176 | 1161 | R1[δ_in_(CH)](80)+R1[ν(CC)](19) |
| 1137 | 1126 | 1141 | - | R2[ν(CC)](36)+γ(C19H_2_)(18)+R2[δ_in_(CH)](11)+ν(C12N)(6)  +ν(C17N)(5) |
| 1109 | 1099 | 1084 | 1087 | R1[ν(CC)](46)+R1[δin(CH](40)+ρ(C12H2)(7)+R1[δin(C12C)](4) |
| 1095 | 1085 | 1079 | 1064 | R2[δin(CH)](48)+R2[ν(CC)](18)+R2[δring](14)+R2[ν(C19N)](8) |
| 1065 | 1056 | 1061 | 1050 | ν(C12N)(49)+[ρ+γ](C19H_2_)(11)+ν(CC)(7)+R2[ν(CC)](6) |
| 1050 | 1040 | - | 1035 | R1[ν(CC)](59)+R1 δ_in_(CH)](19)+R1[δ_trig_](16) |
| 1020 | 1011 | 1003 | 1008 | ρ(C12H_2_)(21)+R1[δ_trig_](18)+R1[ν(CC)](18)+ν(CC)(7)+R1[ν(CC)](7)  +δ(NH)(7)+γ(C12H_2_)(5) |
| 1017 | 1009 | 996 | 1006 | R1[δ_trig_](40)+R1[ν(CC)](24)+ρ(C12H_2_)(12) |
| 1010 | 1002 | 993 | - | R1[oop(CH)](83)+R1[puck](13) |
| 994 | 987 | 983 | 993 | R1[oop(CH)](91)+R1[τ_a_](8) |
| 956 | 949 | 957 | 947 | [ρ+γ+δ](C19H_2_)(48)+R1[oop(CH)](9)+R2[δ_ring_](8)+ν(CC)(8)  +[τ(C19N)](5) |
| 937 | 931 | 919 | 923 | R1[oop(CH)](74)+R1[puck](5)+R1[τ_a_](5) |
| 923 | 917 | 909 | 920 | R2[δ_ring_](55)+R2[ν(CC)](21)+ρ(C19H_2_)(9) |
| 888 | 883 | 870 | 883 | R2[oop(CH)](83)+R2[τ(CC)](15) |
| 863 | 859 | 857 | 843 | ρ(C12H_2_)(18)+R1[oop(CH)](18)+ν(C17N)(10)+ν(CC)(10)  +oop(C=O)(6)+δ(C19H_2_)(6) ν(C12N)(5)+ρ(C19H_2_)(5) |
| 863 | 858 | 856 | 841 | R1[oop(CH)](85) |
| 856 | 852 | - |  | [δ+ν_s_ ](NO_2_)(62)+R2[δ_ring_](9)+R2[ν(N29C)](8) |
| 829 | 824 | 838 | 828 | R1[ν(C12C+ν(CC))](29)+R1[δ_a_+δ_trig_+puck](21)+δ(C12H_2_)(8)  +oop(C=O)(7)+δ(C19H_2_)(6) |
| 825 | 821 | 825 | 812 | oop(C=O)(22)+δ(C19H_2_)(9)+R2[δ_ring_](6)+δ(NO_2_)(5)+ν(CC)(5) |
| 788 | 785 | 792 | 795 | R2[oop(CH)](85)+R2[τ(CC)](8) |
| 768 | 766 | 780 | 779 | R1[oop(CH)](41)+R1[puck](30)+R1[oop(C12C)](10)+R1[δ_a_](5) |
| 749 | 746 | 746 | 746 | oop(NO_2_)(53)+R2[oop (C23N)](27)+R2[τ(CC)](14) |
| 715 | 713 | 698 | 714 | R1[puck](55)+R1[oop(CH)](40) |
| 693 | 691 | 697 | 692 | R2[ν(C19N)](22)+ρ_in_(C=O)(14)+R2[δ_ring_](13)+R1[puck](8) |
| 659 | 658 | - | - | R2[τ(CC)](74)+R2[oop (C23N)](16) |
| 643 | 642 | 647 | - | R2[τ(CC)](36)+R2[oop(C19N+(C23N)](16)+τ(C17N)(18)+oop(NH)(8) |
| 635 | 634 | - | 624 | R1[δ_a_](84) |
| 624 | 623 | 625 | - | R2[τ(CC)](45)+oop(NH)(9)+R2[oop(C19N+C23N)](14)+τ(C17N)(7)+R1[puck](5)+δ(C12H_2_)(5) |
| 607 | 606 | - | 595 | R1[δ_a_+puck+τ_a_](42)+R2[τ(CC)](17)+δ(C12H_2_)(11)+R1[oop(CH)](8) |
| 581 | 581 | - | 572 | δ_in_(NO_2_)(18)+R2[δ_in_(C23N)](14)+oop(NH)(12)+τ(C17N)(9)  +oop(C=O)(9)+ν(CC)(8)+ρin(C=O)(6) |
| 535 | 535 | - | 541 | δ_in_(NO_2_)(19)+oop(C=O)(12)+R2[δ_in_(C19N)](12)+R2[δ_in_(C23N)](11)  +oop(NH)(7) R2[ν(C19N)](6)+R2[ν(CC)](5) |
| 502 | 503 | - | - | R1[τ_a_+δ_a_](40)+R1[oop(C12C)](13)+ρ_in_(C=O)(10)+R1[ν(C12C)](6) |
| 446 | 447 | - | 455 | R2[ν(N29C)](21)+R1[τ_a_](19)+δ(C=O)(12)+R1[oop(C12C)](8)+[τ(C19N)](6)+δ(NO_2_)(6)+ρin(C=O)(6)+R2[δ_ring_](7) |
| 440 | 441 | - | - | R2[ν(N29C)](13)+[τ(C19N)](12)+δ(C=O)(11)+R2[δ_in_(C19N)](11)  +R1[τ_a_](9)+ρ(C19H_2_)(8) δ(NO_2_)(6)+ρ_in_(C=O)(6) |
| 413 | 414 | - | 406 | R1[τ_a_](84)+R1[oop(CH)](15) |

^a^Recommended vibrational normal mode assignments and potential energy distribution (PED)

Types of vibration: ν, stretching; ω, wagging; ρ, rocking; τ, torsion; γ, twisting; δ, deformation (bending), scissoring; oop, out-of-plane bending; in plane bending

Potential energy distribution (contribution$\geq5\%$

**Table S6**. Potential energy distribution of calculated wavenumbers (cm^-1^) for the dimer of benznidazole.

| Unscaled DFT | Scaled | PED^a^(%) |
| --- | --- | --- |
| 3614 | 3433 | ν(N15H)(100) |
| 3519 | 3347 | ν(N46H)(95) |
| 3268 | 3123 | R4[ν(CH)] (99) |
| 3261 | 3116 | R2[ν(CH)](99) |
| 3244 | 3101 | R4[ν(CH)](98) |
| 3244 | 3100 | R2[ν(CH)](99) |
| 3187 | 3050 | R1[ν(CH)](97) |
| 3186 | 3048 | R3[ν(CH)](97) |
| 3178 | 3041 | ν_a_(C50H_2_)(99) |
| 3177 | 3040 | R1[ν(CH)](99) |
| 3176 | 3039 | R3[ν(CH)](100) |
| 3167 | 3031 | R1[ν(CH)](98) |
| 3165 | 3030 | R3[ν(CH)](97) |
| 3159 | 3024 | R1[ν(CH)](99) |
| 3158 | 3023 | R3[ν(CH)](98) |
| 3153 | 3018 | R3[ν(CH)](98) |
| 3152 | 3018 | R1[ν(CH)](96) |
| 3138 | 3005 | ν_a_(C19H_2_)(99) |
| 3110 | 2979 | ν_a_(C12H_2_)(100) |
| 3102 | 2972 | ν_a_(C43H_2_)(99) |
| 3088 | 2959 | ν_s_(C50H_2_)(100) |
| 3081 | 2953 | ν_s_(C19H_2_)(100) |
| 3045 | 2920 | ν_s_(C12H_2_)(99) |
| 3039 | 2915 | ν_s_(C43H_2_)(100) |
| 1734 | 1700 | [δ+ν](C48=O)(56)+ν(C17=O)(17)+ν(C48N)(8) |
| 1722 | 1688 | [ν+δ](C17=O)(57)+[ν+δ] (C48=O)(21)+ν(C17N)(8) |
| 1645 | 1615 | R1[ν(CC)](67)+R1[δ_in_(CH)](23)+R1[δ_a_](10) |
| 1644 | 1614 | R3[ν(CC)](64)+R3[δ_in_(CH)](22)+R3[δ_a_](10) |
| 1625 | 1596 | R1[ν(CC)](68)+R1[δ_in_(CH)](9)+R1[δ](9) |
| 1625 | 1596 | R3[ν(CC)](77)+R3[δ_a_](9)+R3[δ_in_(CH)](9) |
| 1593 | 1566 | ρ_in_(N46H)(39)+ρ_in_ (O18HC)(39)+ν(C48N)(8) |
| 1580 | 1554 | ν_a_(N29O_2_)(72)+δ_in_(N29O_2_)(9)+R2[δ_in_(C23N)](7) |
| 1568 | 1542 | ν_a_(N60O_2_)(51)+ρ_in_(N15H)(10)+ν(C17N)(7)+δin(N60O_2_)(6) |
| 1561 | 1535 | ρ_in_(N15H)(33)+ν(N60O_2_)(23)+ν(C17N)(18) |
| 1530 | 1505 | R1[δ_in_(CH)](59)+R1[ν(CC)](36) |
| 1529 | 1504 | R3[δ_in_(CH)](59)+R3[ν(CC)](32) |
| 1528 | 1503 | R2[ν(CC)](40)+R2[ν(CN)](14)+R2[oop(CH)](14)+R2[δ_in_(CH)](10) |
| 1525 | 1501 | R4[ν(CC)](39)+R4[δ_in_(CH)](23)+R4[ν(CN)](19)+δ(C50H_2_)(6) |
| 1517 | 1493 | R4[ν(CN)](29)+δ(C50H_2_)(25)+R4[δ_in_(CH)](8)+ν_a_(N60O_2_)(8) |
| 1516 | 1492 | R2[ν(CN)](44)+ν(N29O_2_)(9)+R2[oop(CH)](8)+R2[δin(CH)](5) |
| 1507 | 1484 | δ(C12H_2_)(88) |
| 1506 | 1482 | δ(C43H_2_)(93) |
| 1488 | 1465 | δ(C50H_2_)(48)+R4[ν(CN)](24)+R4[δ_in_(CH)](5) |
| 1485 | 1462 | R1[δ_in_(CH)](50)+R1[ν(CC)](33) |
| 1484 | 1462 | R3[δ_in_(CH)](50)+R3[ν(CC)](33) |
| 1470 | 1448 | [δ+ω](C19H_2_)(70)+R2[ν(CN)](6) |
| 1427 | 1406 | R4[ν(CN)](41)+[ω+γ+δ](C50H_2_)(26)+R4[δ_ring_](5) |
| 1423 | 1403 | R2[ν(CN)](55)+[γ+δ](C19H_2_)(16)+R2[δ_ring_](5) |
| 1397 | 1378 | [ω+δ](C19H_2_)(39)+ω(C12H_2_)(27)+ν(C17C)(9) |
| 1391 | 1372 | ω(C43H_2_)(50)+R3[ν(C32C)](5)+R4[ν(CN)](5) |
| 1385 | 1366 | [ν_s_+δ](N60O_2_)(44)+R4[ν(N60C)](17)+ω(C12H_2_+C43H_2_)(11) |
| 1382 | 1363 | ω(C12H_2_)(25)+[ω+δ](C19H_2_)(25) |
| 1378 | 1359 | [ν_s_+δ](N29O_2_)(50)+R2[ν(CN)](12)+R2[ν(N29C)](9)+γ(C19H_2_)(8) |
| 1374 | 1356 | R4[ν(CN)](21)+ω(C50H_2_)(15)+R4[δ_in_(CH)](11)+R4[ν(N60C)](9)+R4[ν(CC)](9) |
| 1372 | 1354 | R2[ν(CN)](38)+R2[ν(N29C)](20)+γ(C19H_2_)(7)+R2[ν(C19N)](5)+R2[δ_ring_](9)  +R2[δ_in_(CH)](5) |
| 1361 | 1343 | R1[δ_in_(CH)](61)+R1[ν(CC)](17)+ω(C12H_2_)(11) |
| 1360 | 1342 | R3[δ_in_(CH)](67)+[ω+γ](C43H_2_)(11) |
| 1351 | 1333 | [ω+γ](C50H_2_)(34)+R4[ν(CN)](18)+R4[δ_ring_](6)+R4[ν(CC)](5) |
| 1339 | 1321 | R3[ν(CC)](72)+[γ+ω](C43H_2_)(12)+R3[δ_in_(CH)](6) R3[δ_in_(CH)](3) |
| 1337 | 1320 | R1[ν(CC)](72)+[γ+ω](C12H_2_)(120+R1[δ_in_(CH)](8) |
| 1319 | 1302 | R2[ν(CN)](19)+R2[δ_in_(CH)](18)+R2[ν(C19N)](16)+ν_s_(N29O_2_)(12)+R2[δ_ring_](8)+γ(C19H_2_)(7)+R2[oop(CH)](7) |
| 1307 | 1291 | [δ+ω+γ](O18HN)(40)+ρ_in_(N46H)(13)+ν(C48N)(13)+ω(C50H_2_)(11) |
| 1284 | 1268 | R4[ν(CN)](17)+[γ+ω+ρ](C50H2)(17)+R4[ν(C50N)](16)+R4[δ_in_(CH)](16)+R4[δ_ring_](7)+δ(O18HC)(5) |
| 1261 | 1246 | ν(C17N)(21)+[γ+ω](C12H_2_)(17)+ω(C19H_2_)(9)+ρin(C17=O)(9)+ν(C12N)(5) |
| 1259 | 1245 | [γ+ρ ](C43H_2_)(47)+δ(O18HC)(11)+R3[ν(CC)](11)+ρ_in_(N46H)(6) |
| 1250 | 1236 | [γ+ρ ](C12H_2_)(47)+ρ_in_(N15H)(18)+R1[ν(CC)](8)+ν(C17N)(5) |
| 1238 | 1224 | γ(C19H_2_)(28)+R2[ν(CC)](10)+R2[δ_ring_](15)+R2[ν(C19N)](8)+R2[ν(N29C)](6) |
| 1229 | 1215 | γ(C50H_2_)(25)+R4[δ_ring_](11)+δ(O18HC)(10)+R4[ν(C50N)](8)+ν(C48N)(5) |
| 1226 | 1212 | R1[ν(C12C)](41)+R1[ν(CC)](21)+R1[δ_trig_](14)+R1[δ_in_(CH](12) |
| 1225 | 1211 | R3[ν(C32C)](38)+R3[ν(CC)](19)+R3[δ_trig_](14)+R3[δ_in_(CH](11) |
| 1207 | 1194 | R1[δ_in_(CH)](77)+R1[ν(CC)](16) |
| 1204 | 1191 | R3[δ_in_(CH)](76)+R3[ν(CC)](14) |
| 1197 | 1184 | R2[ν(CN)](57)+R2[δ_in_(CH)](8)+R2[oop(CH)](7)+R2[δ_ring_](6) |
| 1193 | 1180 | R4[ν(CN)](52)+R4[δ_in_(CH)](18)+R4[δ_ring_](9)+R4[δ_in_(CH)](8) |
| 1183 | 1171 | R1[δ_in_(CH)](76)+R1[ν(CC)](16) |
| 1181 | 1169 | R3[δ_in_(CH)](78)+R3[ν(CC)](16) |
| 1161 | 1150 | R2[ν(CN)](32)+R2[oop(CH)](12)+γ(C19H_2_)(11)+R2[δ_ring_](9) |
| 1140 | 1129 | R4[ν(CN)](22)+R4[ν(CC)](15)+R4[δ_in_(CH)](13)+γ(C50H_2_)(11)+ν_s_(N60O_2_)(7) |
| 1110 | 1100 | R1[ν(CC)](48)+R1[δ_in_(CH)](37)+ρ(C12H_2_)(6) |
| 1109 | 1099 | R3[ν(CC)](46)+R3[δ_in_(CH)](38)+ρ(C43H_2_)(7) |
| 1105 | 1095 | R2[oop(CH)](28)+R2[δ_in_(CH)](24)+R2[ν(CC)](21)+R2[δ_ring_](11)+R2[ν(C19N)](7) |
| 1090 | 1080 | R4[δ_in_(CH)](44)+R4[δ_ring_](14)+R4[ν(CC)](13)+R4[ν(C50N)](8) |
| 1079 | 1070 | ν(C43N)(44)+ρ(C50H_2_)(10)+δ(C48=O)(7) |
| 1065 | 1056 | ν(C12N)(48)+δ(C12H_2_)(7)+δ(N15H)(6)+ρ(C12H_2_)(5) |
| 1050 | 1041 | R1[ν(CC)](54)+R1[δ_in_(CH)](22)+R1[δ_trig_](14) |
| 1049 | 1040 | R3[ν(CC)](57)+R3[δ_in_(CH)](20)+R3[δ_trig_](14) |
| 1023 | 1015 | ρ(C43H_2_)(24)+ν(C48C)(10)+δ(N46H)(9)+δ(O18HC)(8)+γ(C43H2)(6)+ρ_in_(C48=O)(5) |
| 1018 | 1010 | R1[δ_trig_](54)+R1[ν(CC)](35) |
| 1018 | 1010 | R3[δ_trig_](60)+R3[ν(CC)](32) |
| 1016 | 1008 | R1[oop(CH)](72)+R1[puck](13) |
| 1012 | 1004 | [ρ+γ](C12H_2_)(24)+R1[oop(CH)](13)+ν(C17C)(14)+ρ_in_(C17=O)(8)+ν(C12N)(6)  +δ(N15H)(6)+(C12H2)(5) |
| 1010 | 1002 | R3[oop(CH)](81)+R3[puck](13) |
| 999 | 991 | R1[oop(CH)](91)+R1[τ_a_](7) |
| 995 | 988 | R3[oop(CH)](89)+R3[τ_a_](8) |
| 974 | 967 | ρ(C19H_2_)(30)+δ(O18HC)(22)+oop(C17=O)(14) |
| 966 | 959 | [ρ+γ](C50H_2_)(52)+ν(C48C)(9)+τ(C50N)(6) |
| 940 | 934 | R1[oop(CH)](80)+R1[τ_a_](5) |
| 936 | 930 | R3[oop(CH)](73)+R4[δ_ring_](6) |
| 934 | 928 | R4[δ_ring_](52)+R4[ν(CN)](11)+R3[oop(CH)](10)+ρ(C50H_2_)(5) |
| 926 | 920 | R2[δ_ring_](54)+R2[ν(CN)](13) |
| 912 | 907 | [ρ+δ](C12H_2_)(35)+ν(C17C)(17)+ρ_in_(C17=O)(9)+δ(O18HC)(8) |
| 902 | 896 | R4[oop(CH)](25)+ρ(C43H_2_)(18)+ν(C48N)(6)+(C48C)(6)+oop(C48=O)(5)  +δ(C50H_2_)(5) |
| 894 | 888 | R4[oop(CH)](60)+R4[τ(CN)](11)+ρ(C43H_2_)(6) |
| 891 | 886 | R2[oop(CH)](77)+R2[τ(CN)](12) |
| 868 | 863 | R1[oop(CH)](98) |
| 865 | 860 | R3[oop(CH)](97) |
| 858 | 853 | [δ+ν] (N60O_2_)(46)+R4[δ_ring_](10)+oop(C48=O)(9)+R4[ν(N60C)](7)+δ(C50H_2_)(6) |
| 857 | 852 | [δ+ν] (N29O_2_)(64)+R2[δ_ring_](8)+R2[ν(N29C)](8)+R2[ν(C19N)](4) |
| 834 | 830 | oop(C48=O)(14)+δ(C50H_2_)(11)+R3[ν(C32C)](10)+δ(N60O_2_)(10) +R3[ν(CC)](6)+R3[δ_a_](5) |
| 829 | 825 | R1[δ_a_+δ_trig_+puck](21)+R1[ν(C12C)](17)+R1[ν(CC)](14)+ω(C12H_2_)(10)+ν(C17C)(6) |
| 818 | 815 | R4[oop(CH)](23)+oop(C48=O)(14)+R3[ν(C32C)](8)+δ(C50H2)(5) |
| 809 | 806 | R4[oop(CH)](61)+oop(C48=O)(12) |
| 807 | 804 | R2[oop(CH)](73)+R2[τ(CN)](5) |
| 792 | 789 | oop(O18H)(25)+δ(C19H_2_)(11)+R2[δ_ring_](10)+R2[oop(CH)](7)+R2[ν(C19N)](7)  +oop(C17=O)(7) |
| 767 | 764 | R3[oop(CH)](42)+R3[puck](28)+R3[oop(C43C)](10) |
| 766 | 763 | R1[puck](52)+R1[oop(CH)](19)+R1[oop(C12C)](10) |
| 763 | 760 | oop(N60O_2_)(50)+R4[oop(C54N)](23)+R4[τ(CN)](12) |
| 754 | 751 | R2[δ_in_(CH)](59)+R2[oop (C23N)](21)+R2[τ(CN)](15) |
| 717 | 715 | R1[puck](58)+R1[oop(CH)](29)+R1[oop(C12C)](7) |
| 716 | 714 | R3[puck](49)+R3[oop(CH)](27) |
| 705 | 703 | [δ_in_+γ+δ](O18HN)(44)+oop(N46H)(8)+τ(C48N)(7) |
| 704 | 703 | [δ_in_+γ+δ](O18HN)(17)+R4[τ(CN)](11)+R4[oop(C54N)](7) |
| 693 | 692 | [δ_in_+γ](O18H)(50)+oop(N46H)(14)+τ(C47)(14)+τ(C48N)(10) |
| 663 | 662 | R4[τ(CN)](55)+R4[oop(C54N)](14) |
| 661 | 660 | R2[τ(CN)](74)+R2[oop (C23N)](11) |
| 639 | 638 | R4[τ(CN)](65)+R4[oop (C50N)](11) |
| 635 | 634 | R1[δ_a_](65)+R2[τ(CN)](15) |
| 634 | 633 | R3[δ_a_](73)+R4[τ(CN)](8) |
| 632 | 631 | R2[τ(CN)](55)+oop(O18HC)(9)+R2[oop(C19N)](8)+R1[δa](7) |
| 620 | 619 | oop(O18CN)(41)+δ(O18HN)(32)+oop(C17=O)(13) |
| 611 | 611 | oop(N46H)(26)+R3[δ_a_+puck](24)+δ(C43H_2_)(7) |
| 593 | 592 | R1[δ_a_+puck](31)+[δ+oop](O18HC)(22)+δ(C12H_2_)(13) |
| 578 | 577 | [δ_in_+δ+γ](O18HN)(30)+oop(N46H)(11)+oop(C48=O)(10) |
| 554 | 554 | [oop+δ](O18HC)(28)+δ_in_(N29O_2_)(21)+R2[δ_in_ (C23N)](12) |
| 536 | 536 | δ_in_(N60O_2_)(22)+R4[τ(CN)](18)+R4[δ_in_(C54N)](12)+R4[δ_in_(C50N)](11) |
| 516 | 516 | R1[δa](28)+R1[oop(C12C)](10)+oop(C17=O)(9)+oop(N15H)(8)+δ(O18HC)(9) |
| 507 | 508 | R3[τa](36)+R1[oop(C43C)](14)+ρ_in_(C48=O)(7)+δ(C48=O)(5) |
| 491 | 491 | oop(N15H)(22)+τ(O18H)(19)+oop(C17=O)(16) |
| 454 | 455 | R2[ν(N29C)](37)+δ(N29O_2_)(13)+R1[τ_a_](7)+R2[δ_ring_](5) |
| 442 | 443 | R4[ν(N60C)](45)+δ(N60O_2_)(17)+R4[δ_ring_](16) |
| 421 | 422 | R3[τ_a_](22)+δ(C48=O)(19)+δ(O18HN)(16)+R1[oop(C43C)](7)+τ(C50N)(6) |
| 416 | 417 | R1[τ_a_](83)+R1[oop(CH)](15) |
| 415 | 415 | R3[τ_a_] (78)+R3[oop(CH)](15) |

^a^Recommended vibrational normal mode assignments and potential energy distribution (PED)

Types of vibration: ν, stretching; ω, wagging; ρ, rocking; τ, torsion; γ, twisting; δ, deformation (bending), scissoring; oop, out-of-plane bending; in plane bending

Potential energy distribution (contribution$\geq5\%$

**Table S7.** NBO basis of monomer of benznidazole calculated at B3LYP/6−311++G(d,2p) level of theory, with second-order perturbation theory analysis.

| Donor NBO(i) | ED ( i )/e | Acceptor NBO(j) | ED(j)/e | E(2)^a^ kcal/mol | E(j)-E(i)^b^ a.u. | F(i,j)^c^a.u |
| --- | --- | --- | --- | --- | --- | --- |
| π(C1-C2) | 1.665 | π*(C4-C6) | 0.324 | 20.25 | 0.28 | 0.068 |
| π(C1-C2) | 1.665 | π*(C8-C10) | 0.313 | 19.60 | 0.28 | 0.067 |
| π(C4-C6) | 1.666 | π*(C1-C2) | 0.347 | 20.02 | 0.28 | 0.068 |
| π(C4-C6) | 1.666 | π*(C8-C10) | 0.313 | 19.86 | 0.28 | 0.067 |
| π(C8-C10) | 1.665 | π*(C1-C2) | 0.347 | 21.01 | 0.28 | 0.069 |
| π(C8-C10) | 1.665 | π*(C4-C6) | 0.324 | 20.23 | 0.28 | 0.068 |
| σ(N15-H16) | 1.983 | σ*(C17-O18) | 0.014 | 5.22 | 1.26 | 0.072 |
| σ(N22-C27) | 1.979 | σ*(C23-N29) | 0.110 | 5.32 | 1.11 | 0.070 |
| π(C23-N24) | 1.808 | π*(C25-C27) | 0.307 | 20.56 | 0.34 | 0.076 |
| π(C23-N24) | 1.808 | π*(N29-O30) | 0.608 | 15.90 | 0.19 | 0.056 |
| σ(N24-C25) | 1.974 | σ*(C23-N29) | 0.110 | 8.00 | 1.08 | 0.085 |
| σ(C25-C27) | 1.984 | σ*(C19-N22) | 0.029 | 5.03 | 1.02 | 0.064 |
| π(C25-C27) | 1.751 | π*(C23-N24) | 0.458 | 19.06 | 0.26 | 0.067 |
| π(N29-O30) | 1.984 | π*(N29-O30) | 0.608 | 7.00 | 0.33 | 0.051 |
| LP(1)N15 | 1.668 | π*(C17-O18) | 0.314 | 71.79 | 0.27 | 0.124 |
| LP(1)N15 | 1.668 | σ*(C1-C12) | 0.028 | 5.20 | 0.67 | 0.057 |
| LP(2)O18 | 1.861 | σ*(N15-C17) | 0.071 | 22.97 | 0.74 | 0.118 |
| LP(2)O18 | 1.861 | σ*(C17-C19) | 0.073 | 21.16 | 0.59 | 0.101 |
| LP(1)N22 | 1.526 | π*(C23-N24) | 0.458 | 46.96 | 0.27 | 0.101 |
| LP(1)N22 | 1.526 | π*(C25-C27) | 0.307 | 33.13 | 0.29 | 0.092 |
| LP(1)N22 | 1.526 | σ*(C17-C19) | 0.073 | 5.60 | 0.63 | 0.059 |
| LP(1)N24 | 1.912 | σ*(N22-C23) | 0.051 | 9.17 | 0.78 | 0.076 |
| LP(1)N24 | 1.912 | σ*(C25-C27) | 0.018 | 5.08 | 0.94 | 0.063 |
| LP(2)O30 | 1.883 | σ*(N29-O31) | 0.063 | 20.20 | 0.68 | 0.106 |
| LP(2)O30 | 1.883 | σ*(C23-N29) | 0.110 | 13.57 | 0.58 | 0.079 |
| LP(2)O31 | 1.906 | σ*(N29-O30) | 0.608 | 17.78 | 0.76 | 0.105 |
| LP(2)O31 | 1.906 | σ*(C23-N29) | 0.110 | 9.27 | 0.6 | 0.067 |
| LP(3)O31 | 1.519 | π*(N29-O30) | 0.608 | 126.90 | 0.16 | 0.130 |

^a^E(2) is the stabilization energy represented by hyperconjugative interaction.

^b^Energy difference between donor (i) and acceptor (j) NBO orbitals.

^c^F(i,j) is the element of the Fock matrix between NBO orbitals i and j.

**Table S8.** NBO basis of the dimer of benznidazole calculated at B3LYP/6−311++G(d,2p) level of theory, with second-order perturbation theory analysis.

| Donor NBO(i) | ED(i)/e | Acceptor NBO(j) | ED(j)/e | E(2)^a^ kcal/mol | E(j)-E(i)^b^ a.u. | F(i,j)^c^a.u |
| --- | --- | --- | --- | --- | --- | --- |
| π(C1-C2) | 1.673 | π*(C4-C6) | 0.321 | 19.70 | 0.29 | 0.067 |
| π(C1-C2) | 1.673 | π*(C8-C10) | 0.307 | 19.44 | 0.29 | 0.067 |
| π(C4-C6) | 1.666 | π*(C1-C2) | 0.353 | 20.38 | 0.28 | 0.068 |
| π(C4-C6) | 1.666 | π*(C8-C10) | 0.307 | 19.56 | 0.28 | 0.067 |
| π(C8-C10) | 1.661 | π*(C1-C2) | 0.353 | 21.09 | 0.28 | 0.069 |
| π(C8-C10) | 1.661 | π*(C4-C6) | 0.321 | 20.36 | 0.28 | 0.068 |
| σ(N15-H16) | 1.983 | σ*(C17-O18) | 0.046 | 5.10 | 1.20 | 0.070 |
| σ(N22-C27) | 1.979 | σ*(C23-N29) | 0.110 | 5.11 | 1.11 | 0.069 |
| π(C23-N24) | 1.810 | π*(C25-C27) | 0.301 | 20.14 | 0.34 | 0.076 |
| π(C23-N24) | 1.810 | π*(N29-O30) | 0.051 | 15.89 | 0.19 | 0.056 |
| σ(N24-C25) | 1.974 | σ*(C23-N29) | 0.110 | 8.25 | 1.08 | 0.086 |
| σ(C25-C27) | 1.984 | σ*(C19-N22) | 0.025 | 5.22 | 1.03 | 0.066 |
| π(C25-C27) | 1.750 | π*(C23-N24) | 0.450 | 19.27 | 0.26 | 0.067 |
| π(N29-O30) | 1.983 | π*(N29-O30) | 0.051 | 7.49 | 0.32 | 0.053 |
| π(C32-C33) | 1.661 | π*(C35-C37) | 0.322 | 20.19 | 0.28 | 0.068 |
| π(C32-C33) | 1.661 | π*(C39-C41) | 0.318 | 20.05 | 0.28 | 0.067 |
| π(C35-C37) | 1.669 | π*(C39-C41) | 0.318 | 20.17 | 0.28 | 0.068 |
| π(C35-C37) | 1.669 | π*(C32-C33) | 0.340 | 19.84 | 0.29 | 0.067 |
| π(C39-C41) | 1.673 | π*(C32-C33) | 0.340 | 20.42 | 0.29 | 0.069 |
| π(C39-C41) | 1.673 | π*(C35-C37) | 0.322 | 19.83 | 0.28 | 0.067 |
| σ(N46-H47) | 1.982 | σ*(C48-O49) | 0.015 | 5.33 | 1.25 | 0.073 |
| σ(N53-C58) | 1.979 | σ*(C54-N60) | 0.105 | 5.41 | 1.11 | 0.071 |
| π(C54-N55) | 1.812 | π*(C56-C58) | 0.300 | 19.91 | 0.34 | 0.076 |
| π(C54-N55) | 1.812 | π*(N60-O62) | 0.655 | 16.12 | 0.19 | 0.056 |
| σ(N55-C56) | 1.973 | σ*(C54-N60) | 0.105 | 8.24 | 1.08 | 0.086 |
| σ(C56-C58) | 1.984 | σ*(C50-N53) | 0.029 | 5.13 | 1.02 | 0.065 |
| π(C56-C58) | 1.751 | π*(C54-N55) | 0.465 | 18.66 | 0.26 | 0.066 |
| π(N60-O62) | 1.983 | π*(N60-O62) | 0.655 | 7.66 | 0.33 | 0.054 |
| LP(1)N15 | 1.666 | π*(C17-O18) | 0.308 | 56.47 | 0.31 | 0.12 |
| LP(1)N15 | 1.666 | σ*(C12-H14) | 0.017 | 5.72 | 0.66 | 0.06 |
| LP(1)O18 | 1.967 | σ*(N46-H47) | 0.028 | 6.35 | 1.16 | 0.077 |
| LP(2)O18 | 1.873 | σ*(N15-C17) | 0.063 | 21.55 | 0.76 | 0.116 |
| LP(2)O18 | 1.873 | σ*(C17-C19) | 0.073 | 20.56 | 0.61 | 0.101 |
| LP(1)N22 | 1.532 | π*(C23-N24) | 0.499 | 46.98 | 0.27 | 0.101 |
| LP(1)N22 | 1.532 | π*(C25-C27) | 0.301 | 33.22 | 0.29 | 0.092 |
| LP(1)N22 | 1.532 | σ*(C17-C19) | 0.073 | 5.65 | 0.62 | 0.059 |
| LP(1)N24 | 1.913 | σ*(N22-C23) | 0.051 | 9.17 | 0.79 | 0.077 |
| LP(2)O30 | 1.887 | σ*(N29-O31) | 0.060 | 19.62 | 0.69 | 0.105 |
| LP(2)O30 | 1.887 | σ*(C23-N29) | 0.110 | 13.14 | 0.58 | 0.078 |
| LP(2)O31 | 1.901 | σ*(N29-O30) | 0.060 | 18.11 | 0.74 | 0.105 |
| LP(2)O31 | 1.901 | σ*(C23-N29) | 0.110 | 10.11 | 0.6 | 0.07 |
| LP(3)O31 | 1.491 | π*(N29-O30) | 0.630 | 139.97 | 0.15 | 0.133 |
| LP(1)N46 | 1.651 | π*(C48-O49) | 0.331 | 74.67 | 0.26 | 0.126 |
| LP(1)N46 | 1.651 | σ*(C43-H45) | 0.016 | 5.21 | 0.66 | 0.057 |
| LP(2)O49 | 1.866 | σ*(N46-C48) | 0.069 | 22.60 | 0.75 | 0.118 |
| LP(2)O49 | 1.866 | σ*(C48-C50) | 0.068 | 20.53 | 0.59 | 0.100 |
| LP(1)N53 | 1.519 | π*(C54-N55) | 0.465 | 49.20 | 0.27 | 0.103 |
| LP(1)N53 | 1.519 | π*(C56-C58) | 0.300 | 33.37 | 0.29 | 0.092 |
| LP(1)N55 | 1.913 | σ*(N53-C54) | 0.049 | 9.06 | 0.8 | 0.077 |
| LP(2)O61 | 1.897 | σ*(N60-O62) | 0.063 | 18.09 | 0.7 | 0.102 |
| LP(2)O61 | 1.897 | σ*(C54-N60) | 0.105 | 11.65 | 0.6 | 0.074 |
| LP(3)O61 | 1.462 | π*(N60-O62) | 0.655 | 148.12 | 0.15 | 0.135 |
| LP(2)O62 | 1.901 | σ*(N60-O61) | 0.050 | 17.97 | 0.72 | 0.103 |
| LP(2)O62 | 1.901 | σ*(C54-N60) | 0.105 | 10.90 | 0.6 | 0.072 |

^a^E(2) is the stabilization energy represented by hyper conjugative interaction .

^b^Energy difference between donor (i) and acceptor (j) NBO orbitals.

^c^F(i,j) is the element of the Fock matrix between NBO orbitals i and j.

**Table S9.** NBO Hybrid orbitals for atom A and B with their polarization coefficient and percentage of contribution of s and p orbitals in monomer and dimer of benznidazole

| **Bond orbital** | **Hybrid A** | **Ploarization coeficient** | **Atomic orbital (%)** | **Hybrid B** | **Atomic orbital (%)** | **Ploarization coeficient** |
| --- | --- | --- | --- | --- | --- | --- |
| **Monome**r | | | | | | |
| π (C1-C2) | sp^1.00^ | 0.7094 | s(0.00%)p(99.96%) | sp^1.00^ | s(0.00%)p(99.95%) | 0.7048 |
| π (C4-C6) | sp^1.00^ | 0.7064 | s(0.00%)p(99.95%) | sp^1.00^ | s(0.00%)p(99.95%) | 0.7078 |
| π(C8-C10) | sp^1.00^ | 0.7086 | s(0.00%)p(99.95%) | sp^1.00^ | s(0.00%)p(99.95%) | 0.7056 |
| σ(N15-H16) | sp^2.46^ | 0.8463 | s(28.87%)p(71.09%) | sp^0^ | s(99.91%)p(0.09%) | 0.5328 |
| π*(C1-C2) | sp^1.00^ | 0.7048 | s(0.00%)p(99.96%) | sp^1.00^ | s(0.00%)p(99.95%) | -0.7094 |
| π*(C4-C6) | sp^1.00^ | 0.7078 | s(0.00%)p(99.95%) | sp^1.00^ | s(0.00%)p(99.95%) | -0.7064 |
| π*(C23-N24) | sp^1.00^ | 0.7365 | s(0.01%)p(99.89%) | sp^1.00^ | s(0.01%)p(99.81%) | -0.6808 |
| π*(C17-O18) | sp^1.00^ | 0.8409 | s(0.02%)p(99.50%) | sp^1.00^ | s(0.01%)p(99.88%) | -0.5412 |
| π*(N29-O30) | sp^1.00^ | 0.7632 | s(0.04%)p(99.71%) | sp^1.00^ | s(0.03%)p(99.81%) | -0.6462 |
| σ*(C17-O18) | sp^1.00^ | 0.8409 | s(0.02%)p(99.50%) | sp^1.00^ | s(0.01%)p(99.88%) | -0.5412 |
| LP(1)N15 | sp^1.00^ |  | s(0.01%)p(99.97%) |  |  |  |
| LP(1)N22 | sp^1.00^ |  | s(0.00%)p(99.98%) |  |  |  |
| LP(3)O31 | sp^1.00^ |  | s(0.00)%p(99.90%) |  |  |  |
| **Dimer** | | | | | | |
| π (C1-C2) | sp^1.00^ | 0.7127 | s(0.01%)p(99.96%) | sp^1.00^ | s(0.00%)p(99.95%) | 0.7014 |
| π (C4-C6) | sp^1.00^ | 0.7076 | s(0.00%)p(99.95%) | sp^1.00^ | s(0.00%)p(99.95%) | 0.7066 |
| π(C8-C10) | sp^1.00^ | 0.7096 | s(0.00%)p(99.95%) | sp^1.00^ | s(0.00%)p(99.95%) | 0.7046 |
| σ(N15-H16) | sp^2.65^ | 0.8378 | s(27.37%)p(72.58%) | sp^0.00^ | s(99.92%)p(0.00%) | 0.5459 |
| π*(C4-C6) | sp^1.00^ | 0.7066 | s(0.00%)p(99.95%) | sp^1.00^ | s(0.00%)p(99.95%) | -0.7076 |
| π*(C8-C10) | sp^1.00^ | 0.7046 | s(0.00%)p(99.95%) | sp^1.00^ | s(0.00%)p(99.95%) | -0.7096 |
| π*(C17-O18) | sp^40.63^ | 0.8533 | s(2.39%)p(97.10%) | sp^28.69^ | s(3.36%)p(96.54%) | -0.5214 |
| π*(N29-O30) | sp^1.00^ | 0.7695 | s(0.02%)p(99.72%) | sp^1.00^ | s(0.00%)p(99.84%) | -0.6386 |
| π*(N60-O62) | sp^1.00^ | 0.7799 | s(0.47%)p(99.25%) | sp^1.00^ | s(0.33%)p(99.53%) | -0.6259 |
| σ*(C17-O18) | sp^2.29^ | 0.8094 | s(30.37%)p(69.45%) | sp^1.64^ | s(37.87%)p(62.03%) | -0.5872 |
| σ*(N46-H47) | sp^2.40^ | 0.5243 | s(29.37%)p(70.58%) | sp^0.00^ | s(99.91%)p(0.00%) | -0.8515 |
| LP(1)N15 | sp^1.00^ |  | s(0.07%)p(99.91%) |  |  |  |
| LP(1)O18 | sp^0.70^ |  | s(58.74%)p(41.25%) |  |  |  |
| LP(3)O31 | sp^1.00^ |  | s(0.02%)p(99.87%) |  |  |  |
| LP(3)O61 | sp^1.00^ |  | s(0.01%)p(99.88%) |  |  |  |

**Table S10.** The major contribution of electronic transition in UV-Vis spectrum for the monomer and dimer of Benznidazole.

| Molecules | Excited state | Excitation energy (eV) | λ_max_ (nm) | | Oscillator strength (f) | Major contribution (%) |
| --- | --- | --- | --- | --- | --- | --- |
|  |  |  | Calculated | ^a^Ref [67] |  |  |
| Monomer | 1 | 3.27 | 378.88 |  | 0.0010 | HOMO→LUMO (96%) |
|  | 2 | 3.52 | 351.98 |  | 0.0071 | HOMO-1→LUMO (95%) |
|  | 3 | 3.63 | 341.39 |  | 0.0664 | HOMO-2→LUMO (67%) |
|  | 4 | 3.76 | 329.70 | 324 | 0.0461 | HOMO-7→LUMO (11%) |
|  | 6 | 3.99 | 310.01 |  | 0.1522 | HOMO-5→LUMO (35%) |
|  | 9 | 4.97 | 249.24 |  | 0.0118 | HOMO-6→LUMO (91%) |
| Dimer | 1 | 3.29 | 377.21 |  | 0.0000 | HOMO→LUMO (28%) |
|  | 2 | 3.32 | 371.99 |  | 0.0004 | HOMO-1→LUMO (59%) |
|  | 4 | 3.43 | 361.20 |  | 0.0023 | HOMO-1→LUMO+1 (92%) |
|  | 7 | 3.57 | 346.58 |  | 0.1183 | HOMO-4→LUMO (51%) |
|  | 9 | 3.62 | 342.08 |  | 0.0184 | HOMO-4→LUMO (17%) |
|  | 13 | 3.83 | 323.18 |  | 0.0773 | HOMO-14→LUMO+1 (13%) |
|  | 14 | 3.89 | 317.94 |  | 0.0516 | HOMO-6→LUMO (16%) |
|  | 15 | 3.91 | 317.01 |  | 0.0759 | HOMO-7→LUMO (27%) |

^a^Ref. [67]

**Table S11.** Local reactivity descriptors for the monomer of benznidazole calculated at B3LYP/6−311++G(d,2p) level of theory.

| **Atom no** | **f_k_^+^** | **s_k_^+^** | **w_k_^+^** | **f_k_^–^** | **s_k_^–^** | **w_k_^–^** | **f_k_^0^** | **s_k_^0^** | **w_k_^0^** |
| --- | --- | --- | --- | --- | --- | --- | --- | --- | --- |
| C1 | 0.1906 | 0.0492 | 1.3200 | −0.0195 | −0.0050 | −0.1350 | 0.0538 | 0.0139 | 0.3726 |
| C2 | 0.1112 | 0.0287 | 0.7702 | −0.0002 | −0.0001 | −0.0017 | −0.1436 | −0.0371 | −0.9944 |
| H3 | −0.0891 | −0.0230 | −0.6168 | −0.0009 | −0.0002 | −0.0061 | 0.1581 | 0.0408 | 1.0948 |
| C4 | 0.1442 | 0.0372 | 0.9985 | 0.0071 | 0.0018 | 0.0494 | −0.1273 | −0.0329 | −0.8812 |
| H5 | −0.0898 | −0.0232 | −0.6216 | 0.0087 | 0.0022 | 0.0600 | 0.1562 | 0.0403 | 1.0817 |
| C6 | 0.2855 | 0.0737 | 1.9773 | 0.0161 | 0.0042 | 0.1115 | −0.0660 | −0.0170 | −0.4571 |
| H7 | −0.0943 | −0.0244 | −0.6532 | 0.0103 | 0.0027 | 0.0716 | 0.1532 | 0.0396 | 1.0607 |
| C8 | 0.0919 | 0.0237 | 0.6365 | 0.0085 | 0.0022 | 0.0587 | −0.1522 | −0.0393 | −1.0535 |
| H9 | −0.0883 | −0.0228 | −0.6117 | 0.0088 | 0.0023 | 0.0606 | 0.1566 | 0.0404 | 1.0843 |
| C10 | 0.1575 | 0.0407 | 1.0905 | −0.0017 | −0.0005 | −0.0120 | −0.1151 | −0.0297 | −0.7966 |
| H11 | −0.1009 | −0.0261 | −0.6989 | −0.0026 | −0.0007 | −0.0183 | 0.1674 | 0.0432 | 1.1590 |
| C12 | 0.0862 | 0.0222 | 0.5966 | 0.0008 | 0.0002 | 0.0054 | −0.1562 | −0.0403 | −1.0816 |
| H13 | −0.1053 | −0.0272 | −0.7293 | 0.0066 | 0.0017 | 0.0460 | 0.1760 | 0.0454 | 1.2188 |
| H14 | −0.0897 | −0.0232 | −0.6209 | 0.0061 | 0.0016 | 0.0420 | 0.1531 | 0.0395 | 1.0601 |
| N15 | 0.3528 | 0.0911 | 2.4428 | 0.0169 | 0.0044 | 0.1173 | −0.4565 | −0.1179 | −3.1608 |
| H16 | −0.2023 | −0.0522 | −1.4008 | −0.0191 | −0.0049 | −0.1325 | 0.3235 | 0.0835 | 2.2404 |
| C17 | −0.3378 | −0.0872 | −2.3392 | −0.0107 | −0.0028 | −0.0742 | 0.4959 | 0.1280 | 3.4338 |
| O18 | 0.4359 | 0.1125 | 3.0183 | 0.0414 | 0.0107 | 0.2870 | −0.4334 | −0.1119 | −3.0012 |
| C19 | 0.1340 | 0.0346 | 0.9279 | −0.0067 | −0.0017 | −0.0463 | −0.1707 | −0.0441 | −1.1817 |
| H20 | −0.1110 | −0.0287 | −0.7684 | −0.0034 | −0.0009 | −0.0234 | 0.1871 | 0.0483 | 1.2958 |
| H21 | −0.1036 | −0.0268 | −0.7173 | 0.0283 | 0.0073 | 0.1959 | 0.1593 | 0.0411 | 1.1032 |
| N22 | 0.2010 | 0.0519 | 1.3916 | 0.0589 | 0.0152 | 0.4079 | −0.3156 | −0.0815 | −2.1854 |
| C23 | −0.1487 | −0.0384 | −1.0296 | −0.0180 | −0.0047 | −0.1248 | 0.3893 | 0.1005 | 2.6956 |
| N24 | 0.2060 | 0.0532 | 1.4261 | 0.1018 | 0.0263 | 0.7052 | −0.3725 | −0.0962 | −2.5795 |
| C25 | 0.1252 | 0.0323 | 0.8672 | 0.0310 | 0.0080 | 0.2145 | 0.0007 | 0.0002 | 0.0051 |
| H26 | −0.0972 | −0.0251 | −0.6729 | 0.0351 | 0.0091 | 0.2433 | 0.1452 | 0.0375 | 1.0051 |
| C27 | 0.0494 | 0.0128 | 0.3421 | 0.1142 | 0.0295 | 0.7907 | −0.0391 | −0.0101 | −0.2709 |
| H28 | −0.1075 | −0.0278 | −0.7445 | 0.0308 | 0.0080 | 0.2133 | 0.1576 | 0.0407 | 1.0913 |
| N29 | −0.2407 | −0.0622 | −1.6670 | 0.1230 | 0.0318 | 0.8519 | 0.2821 | 0.0728 | 1.9534 |
| O30 | 0.1936 | 0.0500 | 1.3404 | 0.2152 | 0.0556 | 1.4898 | −0.3279 | −0.0847 | −2.2705 |
| O31 | 0.2412 | 0.0623 | 1.6704 | 0.2133 | 0.0551 | 1.4768 | −0.4393 | −0.1134 | −3.0417 |

**Table S12.** Local reactivity descriptors for the dimer of benznidazole calculated at B3LYP/6−311++G(d,2p) level of theory.

| **Atom no** | **f_k_^+^** | **s_k_^+^** | **w_k_^+^** | **f_k_^–^** | **s_k_^–^** | **w_k_^–^** | **f_k_^0^** | **s_k_^0^** | **w_k_^0^** |
| --- | --- | --- | --- | --- | --- | --- | --- | --- | --- |
| C1 | 0.0491 | 0.0135 | 0.3419 | −0.0125 | −0.0034 | −0.0873 | −0.0309 | −0.0085 | −0.2150 |
| C2 | −0.0021 | −0.0006 | −0.0144 | 0.0060 | 0.0016 | 0.0416 | −0.2036 | −0.0558 | −1.4180 |
| H3 | 0.0111 | 0.0030 | 0.0774 | 0.0043 | 0.0012 | 0.0299 | 0.2071 | 0.0568 | 1.4424 |
| C4 | 0.0415 | 0.0114 | 0.2891 | 0.0118 | 0.0032 | 0.0823 | −0.1822 | −0.0499 | −1.2685 |
| H5 | 0.0148 | 0.0041 | 0.1033 | 0.0096 | 0.0026 | 0.0669 | 0.2089 | 0.0573 | 1.4547 |
| C6 | 0.0658 | 0.0180 | 0.4582 | 0.0131 | 0.0036 | 0.0914 | −0.1716 | −0.0470 | −1.1948 |
| H7 | 0.0145 | 0.0040 | 0.1012 | 0.0097 | 0.0026 | 0.0673 | 0.2089 | 0.0573 | 1.4550 |
| C8 | −0.0001 | 0.0000 | −0.0010 | 0.0042 | 0.0011 | 0.0290 | −0.1936 | −0.0531 | −1.3481 |
| H9 | 0.0153 | 0.0042 | 0.1066 | 0.0035 | 0.0010 | 0.0245 | 0.2136 | 0.0585 | 1.4873 |
| C10 | 0.0331 | 0.0091 | 0.2304 | −0.0071 | −0.0019 | −0.0492 | −0.1749 | −0.0480 | −1.2183 |
| H11 | 0.0059 | 0.0016 | 0.0411 | −0.0201 | −0.0055 | −0.1402 | 0.2365 | 0.0648 | 1.6468 |
| C12 | −0.0076 | −0.0021 | −0.0530 | 0.0002 | 0.0001 | 0.0017 | −0.1983 | −0.0543 | −1.3807 |
| H13 | 0.0045 | 0.0012 | 0.0313 | −0.0054 | −0.0015 | −0.0377 | 0.2371 | 0.0650 | 1.6514 |
| H14 | 0.0072 | 0.0020 | 0.0498 | 0.0100 | 0.0027 | 0.0694 | 0.2015 | 0.0552 | 1.4035 |
| N15 | 0.0117 | 0.0032 | 0.0812 | 0.0119 | 0.0033 | 0.0828 | −0.5970 | −0.1636 | −4.1574 |
| H16 | 0.0090 | 0.0025 | 0.0623 | 0.0070 | 0.0019 | 0.0484 | 0.3948 | 0.1082 | 2.7496 |
| C17 | 0.0027 | 0.0007 | 0.0185 | −0.0106 | −0.0029 | −0.0737 | 0.7037 | 0.1929 | 4.9004 |
| O18 | 0.0054 | 0.0015 | 0.0379 | −0.0108 | −0.0030 | −0.0749 | −0.6949 | −0.1905 | −4.8393 |
| C19 | −0.0014 | −0.0004 | −0.0096 | −0.0027 | −0.0007 | −0.0185 | −0.2466 | −0.0676 | −1.7173 |
| H20 | 0.0090 | 0.0025 | 0.0624 | 0.0104 | 0.0029 | 0.0726 | 0.2329 | 0.0638 | 1.6218 |
| H21 | 0.0077 | 0.0021 | 0.0537 | −0.0024 | −0.0007 | −0.0168 | 0.2518 | 0.0690 | 1.7532 |
| N22 | −0.0008 | −0.0002 | −0.0057 | 0.0239 | 0.0065 | 0.1662 | −0.3992 | −0.1094 | −2.7801 |
| C23 | 0.0162 | 0.0044 | 0.1129 | −0.0058 | −0.0016 | −0.0404 | 0.4703 | 0.1289 | 3.2750 |
| N24 | 0.0074 | 0.0020 | 0.0517 | 0.0537 | 0.0147 | 0.3737 | −0.4494 | −0.1232 | −3.1296 |
| C25 | 0.0270 | 0.0074 | 0.1882 | 0.0190 | 0.0052 | 0.1325 | −0.0456 | −0.0125 | −0.3175 |
| H26 | 0.0105 | 0.0029 | 0.0732 | 0.0178 | 0.0049 | 0.1242 | 0.2065 | 0.0566 | 1.4377 |
| C27 | 0.0206 | 0.0057 | 0.1436 | 0.0358 | 0.0098 | 0.2491 | −0.0129 | −0.0035 | −0.0898 |
| H28 | 0.0060 | 0.0016 | 0.0417 | 0.0015 | 0.0004 | 0.0102 | 0.2410 | 0.0661 | 1.6781 |
| N29 | −0.0038 | −0.0011 | −0.0267 | 0.0566 | 0.0155 | 0.3942 | 0.4315 | 0.1183 | 3.0050 |
| O30 | 0.0109 | 0.0030 | 0.0756 | 0.1122 | 0.0308 | 0.7814 | −0.3925 | −0.1076 | −2.7336 |
| O31 | 0.0075 | 0.0021 | 0.0522 | 0.0993 | 0.0272 | 0.6917 | −0.4675 | −0.1281 | −3.2553 |
| C32 | 0.0802 | 0.0220 | 0.5584 | −0.0094 | −0.0026 | −0.0654 | −0.0062 | −0.0017 | −0.0429 |
| C33 | 0.0043 | 0.0012 | 0.0301 | −0.0068 | −0.0019 | −0.0472 | −0.1945 | −0.0533 | −1.3543 |
| H34 | 0.0162 | 0.0044 | 0.1127 | −0.0137 | −0.0038 | −0.0955 | 0.2300 | 0.0631 | 1.6019 |
| C35 | 0.0428 | 0.0117 | 0.2982 | 0.0013 | 0.0004 | 0.0091 | −0.1727 | −0.0473 | −1.2025 |
| H36 | 0.0193 | 0.0053 | 0.1343 | 0.0030 | 0.0008 | 0.0209 | 0.2158 | 0.0591 | 1.5027 |
| C37 | 0.0985 | 0.0270 | 0.6857 | 0.0110 | 0.0030 | 0.0767 | −0.1606 | −0.0440 | −1.1182 |
| H38 | 0.0179 | 0.0049 | 0.1247 | 0.0091 | 0.0025 | 0.0634 | 0.2080 | 0.0570 | 1.4484 |
| C39 | 0.0009 | 0.0002 | 0.0060 | 0.0101 | 0.0028 | 0.0706 | −0.2034 | −0.0557 | −1.4163 |
| H40 | 0.0215 | 0.0059 | 0.1497 | 0.0087 | 0.0024 | 0.0603 | 0.2095 | 0.0574 | 1.4588 |
| C41 | 0.0449 | 0.0123 | 0.3125 | 0.0018 | 0.0005 | 0.0125 | −0.1721 | −0.0472 | −1.1987 |
| H42 | 0.0143 | 0.0039 | 0.0997 | 0.0022 | 0.0006 | 0.0153 | 0.2127 | 0.0583 | 1.4815 |
| C43 | −0.0161 | −0.0044 | −0.1118 | 0.0012 | 0.0003 | 0.0084 | −0.2098 | −0.0575 | −1.4609 |
| H44 | 0.0178 | 0.0049 | 0.1237 | 0.0069 | 0.0019 | 0.0483 | 0.2400 | 0.0658 | 1.6710 |
| H45 | 0.0174 | 0.0048 | 0.1215 | −0.0044 | −0.0012 | −0.0305 | 0.2148 | 0.0589 | 1.4958 |
| N46 | 0.0347 | 0.0095 | 0.2415 | 0.0050 | 0.0014 | 0.0351 | −0.6072 | −0.1664 | −4.2283 |
| H47 | 0.0072 | 0.0020 | 0.0503 | −0.0060 | −0.0016 | −0.0415 | 0.4359 | 0.1195 | 3.0357 |
| C48 | 0.0016 | 0.0005 | 0.0114 | −0.0086 | −0.0023 | −0.0595 | 0.6620 | 0.1815 | 4.6100 |
| O49 | 0.0898 | 0.0246 | 0.6255 | 0.0206 | 0.0056 | 0.1435 | −0.6136 | −0.1682 | −4.2727 |
| C50 | 0.0032 | 0.0009 | 0.0221 | −0.0034 | −0.0009 | −0.0240 | −0.2363 | −0.0648 | −1.6457 |
| H51 | 0.0035 | 0.0010 | 0.0245 | −0.0075 | −0.0020 | −0.0519 | 0.2498 | 0.0685 | 1.7397 |
| H52 | 0.0131 | 0.0036 | 0.0912 | 0.0182 | 0.0050 | 0.1265 | 0.2240 | 0.0614 | 1.5602 |
| N53 | −0.0048 | −0.0013 | −0.0334 | 0.0320 | 0.0088 | 0.2226 | −0.3968 | −0.1088 | −2.7634 |
| C54 | 0.0070 | 0.0019 | 0.0484 | −0.0205 | −0.0056 | −0.1426 | 0.4721 | 0.1294 | 3.2875 |
| N55 | 0.0149 | 0.0041 | 0.1035 | 0.0493 | 0.0135 | 0.3430 | −0.4605 | −0.1262 | −3.2069 |
| C56 | 0.0229 | 0.0063 | 0.1595 | 0.0202 | 0.0055 | 0.1405 | −0.0487 | −0.0133 | −0.3390 |
| H57 | 0.0119 | 0.0033 | 0.0831 | 0.0218 | 0.0060 | 0.1517 | 0.2044 | 0.0560 | 1.4236 |
| C58 | 0.0052 | 0.0014 | 0.0359 | 0.0607 | 0.0166 | 0.4227 | −0.0375 | −0.0103 | −0.2610 |
| H59 | 0.0011 | 0.0003 | 0.0074 | 0.0193 | 0.0053 | 0.1341 | 0.2355 | 0.0646 | 1.6403 |
| N60 | 0.0004 | 0.0001 | 0.0025 | 0.0772 | 0.0212 | 0.5377 | 0.4281 | 0.1173 | 2.9814 |
| O61 | 0.0117 | 0.0032 | 0.0818 | 0.1205 | 0.0330 | 0.8389 | −0.4417 | −0.1211 | −3.0762 |
| O62 | −0.0017 | −0.0005 | −0.0119 | 0.1362 | 0.0373 | 0.9482 | −0.4667 | −0.1279 | −3.2502 |

**Table S13.** Benznidazole’s specific heat, enthalpy, and entropy at temperatures ranging from 50 to 850 K calculated at the B3LYP/6−311++G(d,2p) level of theory.

| Temperature  (K) | Enthalpy  (kcal/mol) | Sp.Heat  (cal/mol−K) | Entropy  (cal/mol−K) |
| --- | --- | --- | --- |
| 50 | 150.2610 | 18.3410 | 74.6860 |
| 100 | 151.3670 | 25.8370 | 91.1060 |
| 150 | 152.8470 | 33.4400 | 103.8040 |
| 200 | 154.7260 | 41.8930 | 115.1320 |
| 250 | 157.0490 | 51.1150 | 125.9030 |
| 300 | 159.8430 | 60.6630 | 136.4280 |
| 350 | 163.1130 | 70.0590 | 146.7960 |
| 400 | 166.8400 | 78.9500 | 157.0030 |
| 450 | 170.9960 | 87.1380 | 167.0170 |
| 500 | 175.5410 | 94.5560 | 176.7980 |
| 550 | 180.4500 | 101.2220 | 186.7840 |
| 600 | 185.6630 | 107.1880 | 196.0260 |
| 650 | 191.1580 | 112.5320 | 204.9790 |
| 700 | 196.9070 | 117.3320 | 213.6450 |
| 750 | 202.8840 | 121.6570 | 222.0270 |
| 800 | 209.0660 | 125.5680 | 230.1330 |
| 850 | 215.4350 | 129.1190 | 237.9750 |
